# Metallic Micro‐Nano Network‐Based Soft Transparent Electrodes: Materials, Processes, and Applications

**DOI:** 10.1002/advs.202302858

**Published:** 2023-10-27

**Authors:** Liyang Chen, Arshad Khan, Shuqin Dai, Amine Bermak, Wen‐Di Li

**Affiliations:** ^1^ Department of Mechanical Engineering University of Hong Kong Hong Kong 00000 China; ^2^ Department of Information Technology and Electrical Engineering ETH Zurich Zurich 8092 Switzerland; ^3^ Division of Information and Computing Technology College of Science and Engineering Hamad Bin Khalifa University Doha 34110 Qatar; ^4^ Department School of Electrical and Electronic Engineering Nanyang Technological University Singapore 639798 Singapore

**Keywords:** flexible electronic devices, metallic micro–nano networks, soft transparent electrodes, transparent bio‐interfaces, wearable sensors

## Abstract

Soft transparent electrodes (TEs) have received tremendous interest from academia and industry due to the rapid development of lightweight, transparent soft electronics. Metallic micro‐nano networks (MMNNs) are a class of promising soft TEs that exhibit excellent optical and electrical properties, including low sheet resistance and high optical transmittance, as well as superior mechanical properties such as softness, robustness, and desirable stability. They are genuinely interesting alternatives to conventional conductive metal oxides, which are expensive to fabricate and have limited flexibility on soft surfaces. This review summarizes state‐of‐the‐art research developments in MMNN‐based soft TEs in terms of performance specifications, fabrication methods, and application areas. The review describes the implementation of MMNN‐based soft TEs in optoelectronics, bioelectronics, tactile sensors, energy storage devices, and other applications. Finally, it presents a perspective on the technical difficulties and potential future possibilities for MMNN‐based TE development.

## Graphical abstract

This article comprehensively summarizes the latest advancements in the field of MMNN‐based soft TEs, focusing on their fabrication techniques, performance characteristics, and application domains. The review provides insights into the integration of MMNN‐based soft TEs within various areas, including optoelectronics, bioelectronics, tactile sensors, and energy storage devices, among others. It also outlines challenges and future prospects for MMNN‐based TEs advancement.

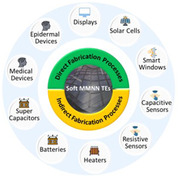

## 1 Introduction

In recent years, considerable advancements have been made in soft electronics, a developing field of electronic technology.^[^ ^1^, ^2^ ^]^ It has superior capabilities to conventional silicon‐based electronics in terms of bendability, stretchability, foldability, conformal attachability, light weight, and compatibility with large‐area manufacturing techniques like roll‐to‐roll (R2R).^[^ ^3^ ^]^ It offers a wide range of applications, including soft solar cells,^[^ ^4^ ^]^ soft displays,^[^ ^5^ ^]^ soft transparent heaters,^[^ ^6^ ^]^ soft touchscreens,^[^ ^7^ ^]^ soft sensors,^[^ ^8^, ^9^ ^]^ soft energy storage devices,^[^ ^10^, ^11^ ^]^ and smart windows. Due to its conformal attachability to human skin and biocompatibility with surgical implements, it also enables a number of other applications in modern medicine.^[^ ^12^, ^13^, ^14^ ^]^

Soft TEs are essential components of the aforementioned soft electronic devices, serving as transporters of charge to or collectors of charge from nearby functional layers. Additionally, they enable light to pass through or leave these devices. A diversity of materials has been exploited as soft TEs, which can be briefly classified into four basic categories: conventional transparent conductive oxides (TCOs), carbon‐based materials, conductive polymers, and metallic micro‐nano networks (MMNNs). Indium tin oxide (ITO), a representative of TCOs, has dominated the TEs market for decades. However, due to improper post‐sintering treatment, poor bending strain tolerance, and cyclic flexibility resulting from the inherent brittleness of ITO, as well as the high cost of the scarce indium element, flexible ITO‐coated plastic (ITO/PET) electrodes have significantly lower conductivity than their rigid counterpart.^[^ ^15^ ^]^ Carbon‐based materials, mostly referred to as carbon nanotubes (CNTs) ^[^ ^16^ ^]^ and graphene,^[^ ^17^ ^]^ can be extremely transparent with only a few layers, although they have limited electrical conductivity. Additionally, the quality of carbon‐based materials is relatively resistant to manufacturing processes and inherent material characteristics. Conductive polymers, such as poly(3,4‐ethylenedioxythiophene polystyrene sulfonate (PEDOT:PSS),^[^ ^15^ ^]^ are an alternative choice for soft TEs, but their poor long‐term stability prevents them from being used in real applications.

Given their superior electrical conductivity, stability, and abundance (low cost) on Earth, metals are the material of choice among all conductive materials. The developed metallic electrodes have high optical transparency, high electrical and thermal conductivity, and high flexibility, all of which are favorable attributes when fabricating electronic devices on broad sheets of soft substrate materials.^[^ ^18^ ^]^ Ultra‐thin metal film‐based soft TEs exhibit modest optical transmittance and electrical conductivity, if the fabrication process is carefully carried out to prevent the creation of metal islands.^[^ ^4^, ^19^ ^]^ The development of metal oxide/metal/metal oxide and dielectric/metal/dielectric multilayer structures enhances metallic soft TEs' optoelectronic capabilities, but it requires careful design, exacting experimental control, and expensive machinery.^[^ ^19^, ^20^, ^21^, ^22^ ^]^ Additionally, it is difficult to fabricate these uniform, ultra‐thin metal films over a large substrate, therefore considerable advancements in fabrication methods must be made before these can be considered leading TEs contenders for soft electronics. MMNN‐based TEs, developed with the advancement of micro/nanofabrication technologies, are currently the most competitive candidate for soft TEs with high optoelectronic performances, exceptional flexibility, desired cost efficiency, and fabrication scalability.^[^ ^1^, ^2^ ^]^ Additionally, MMNN‐based TEs' electrical conductivity, optical transmittance, work functions, and chemical characteristics can all be individually tuned on demand by modifying their geometrical features and choosing the right metallic material from a wider range of options.^[^ ^6^ ^]^

Despite this potential, there are still certain general issues with the geometric structures and fabrication methods of MMNN‐based TEs that limit their commercial success. For instance, the physical deposition of metal parts from the vapor phase is typically required to fabricate MMNN‐based TEs, which is an expensive process that uses vacuum‐based manufacturing.^[^ ^4^, ^23^ ^]^ In many applications, a thick layer of metal network on the substrate is necessary to get a high enough conductivity, but this creates a rough TE surface that adversely affects the performance of multi‐layer soft electronic devices.^[^ ^24^, ^25^ ^]^ Similar to this, poor reliability is caused by the metal's poor adherence to the substrate surface, which is especially evident in highly flexible optoelectronic devices.^[^ ^26^ ^]^ Additionally, MMNN TEs suffer chemical and thermal instabilities. To make MMNN‐based TEs more commercially viable, numerous researchers are tackling these challenges. The work on flexible TEs has been reviewed in several articles that were recently published. Lu et al. summarized the major problems encountered by metal‐based TEs and reviewed the associated research strategies and notable developments in recent years.^[^ ^27^ ^]^ Yang et al.^[^ ^28^ ^]^ and Zhang et al.^[^ ^29^ ^]^ recently reviewed the progress of soft TEs based on metallic micro‐nano structures and their use in organic and perovskite solar cells. Lee et al. summarized the technological advancements of metal mesh‐based TEs and their implementations in organic optoelectronic devices, such as organic LEDs, organic and perovskite solar cells, supercapacitors, and electrochromic devices.^[^ ^1^ ^]^ Lu et al. discussed recent works on metal‐based TEs in photovoltaics and provided information on anticipated future developments.^[^ ^30^ ^]^ Zhu et al. performed a thorough review of current developments in soft Ag nanowire (NW)‐based TEs in terms of performance requirements, material synthesis, fabrication procedures, performance improvements, and potential applications.^[^ ^31^ ^]^ Sannicolo et al. reviewed the developments in the major applications reported for MMNN‐based TEs and offered suggestions on how to increase both the stability and usability of these TEs.^[^ ^32^ ^]^ All of these reviews came to the same conclusion: MMNN‐based TEs might be a promising replacement for traditional ITO electrodes.

This article reviews recent developments in the area of MMNN soft TEs, discussing performance evaluation, fabrication strategies, and novel applications. It also gives a general overview of the main challenges and opportunities facing MMNN soft TEs as they move closer to commercialization. Specifically, the general performance evaluation criteria of soft TEs including optoelectronic performance, mechanical stability, and environmental stability are introduced in Section 2. A comprehensive review of fabrication methods of MMNN soft TEs is presented in Section 3, divided into direct (Section 3.1) and indirect (Section 3.2) pattern formation approaches of MMNNs, together with transfer techniques of the network to flexible transparent substrates (Section 3.3). Overall, the systematic “patterning‐metallization‐transfer” workflow is a universal fabrication strategy for producing soft TEs. Various applications of MMNN soft TEs are discussed in Section 4, comprising optoelectronics, bioelectronics, sensors, energy storage devices, and other applications. Finally, an outlook of the main challenges and prospects of MMNN soft TEs towards commercialization is discussed.

## 2 MMNN Materials

### 2.1 Metallic Micro‐Nano Networks Materials

MNWNs have gained popularity as a preferred choice for constructing MMNN‐based soft electrodes. Among MNWNs, Ag is the most common material due to its excellent electrical conductivity and the availability of established recipes for Ag NW suspensions.^[^ ^33^ ^]^ However, Ag NWs often require additional passivation due to their poor thermal, electrical, and chemical stability. Various encapsulation methods, such as such as oxynitride,^[^ ^34^ ^]^ MXene,^[^ ^35^ ^]^ propolis,^[^ ^36^ ^]^ have been explored for this purpose. In addition to Ag NWs, copper (Cu) and gold (Au) MNWNs are also under investigation. Cu offers excellent electrical conductivity at a significantly lower cost than Ag and Au. However, Cu NWs are unstable in air, and their resistance increases more rapidly compared to Ag NWs. To address this issue, Cu‐core/nickel (Ni)‐shell NWs have been developed to achieve higher stability. Yet, their electrical conductivity is compromised due to Ni's weaker conductance.^[^ ^37^ ^]^ More recently, researchers have made advancements by developing reduced graphene oxide‐coated Cu NWs, which exhibit superior optoelectronic performance and long‐term electrical stability, showing promise for device‐level applications.^[^ ^38^ ^]^ On the other hand, Au NWs present a competitive alternative for various applications due to their high conductance, chemical inertness, environmental stability, and biocompatibility.^[^ ^12^, ^39^ ^]^ Their work function is close to the highest occupied molecular orbital (HOMO) levels of many p‐type organic semiconductors, making them particularly ideal for serving as the anode in organic devices with low hole injection barriers and contact resistance. However, the overall performance of Au NW networks as soft TEs, in terms of optical transparency and electrical conductivity, still lags behind that of Ag NW networks.^[^ ^10^, ^11^, ^40^ ^]^ Manufacturing metallic NW‐based electrodes can be achieved through spin‐coating or screen printing, which are relatively simple methods but offer limited control over the NW distribution, resulting in a high haze factor. Alternatively, uniformly aligned Ag NW microgrids with a lower haze factor can be produced, albeit through a more complex fabrication process.^[^ ^41^ ^]^

Metallic nanoparticles (NPs) also hold significant popularity as materials for MMNN‐based soft electrodes, often utilized in the form of NP inks and processed through printing or laser direct writing (LDW). Among metallic NPs, Cu, Au, and Ag NP inks show promise for printing metallic microstructures. Cu NP inks, known for their low cost and strong electrical conductivity, are highly desired for printing electronics. However, their vulnerability to corrosion necessitates protective shells, which limits their applications.^[^ ^42^ ^]^ On the other hand, Au NP inks exhibit the best corrosion resistance among metals,^[^ ^42^ ^]^ but their usage in industrial applications is restricted due to relatively high electrical resistance, cost, and the release of volatile organic compounds during fabrication.^[^ ^43^ ^]^ As a result, Ag NP inks dominate the market for printing, owing to their reasonable price, excellent conductivity, and acceptable stability.^[^ ^44^ ^]^

In addition to MMNWs and metallic NPs, bulk metals, metal ion solutions, and liquid metals offer alternatives for MMNN soft electrodes. High‐purity bulk metals are utilized as the source for vacuum deposition, leading to dense metal layers with a thickness usually less than 100 nm. Metal ion solutions, on the other hand, are employed for electroplating and electroless plating to deposit metal layers generally a few microns thick.^[^ ^2^ ^]^ The fabrication process of MMNNs with bulk metals and metal ion solutions involves combining lithography methods for micropattern generation. In contrast, liquid metal can be patterned by both indirect approaches (combination with lithography methods)^[^ ^45^ ^]^ and direct printing.^[^ ^46^ ^]^ Researchers have successfully developed visually imperceptible liquid metal micro‐meshes using LDW^[^ ^47^ ^]^ and inkjet printing.^[^ ^48^ ^]^ Compared to solid‐state MMNNs, liquid metal soft electrodes exhibit comparable optoelectronic performance and significantly better flexibility and stretchability. However, their costly ingredients currently limit their commercialization potential.

### 2.2 Soft Substrate Materials

Various polymers find application as flexible substrates for MMNN soft electrodes, offering diverse properties to cater to specific needs. Among these, polyethylene terephthalate (PET) stands out as the most widely used choice due to its exceptional attributes, including high transparency, good flexibility, reasonable chemical resistance, non‐toxicity, and low cost.^[^ ^49^ ^]^ However, when special properties are required, alternative polymers come into play. For instance, polyimide (PI) exhibits superior thermal and chemical stability compared to PET, making it the preferred option for high‐temperature operation or harsh environments.^[^ ^50^, ^51^ ^]^ In the realm of stretchable electronics, elastomer substrates like polydimethylsiloxane (PDMS) or polyurethane are commonly employed.^[^ ^52^ ^]^ PDMS offers advantages such as high optical transparency, good chemical resistance, excellent biocompatibility, and ease of processing. On the other hand, the properties of polyurethane can vary depending on its formulation and additives. In certain cases, polyurethane may have a smaller Young's modulus than PDMS, thereby providing enhanced stretchability.^[^ ^53^ ^]^

## 3 Fabrication Methods

**Figure 1:**
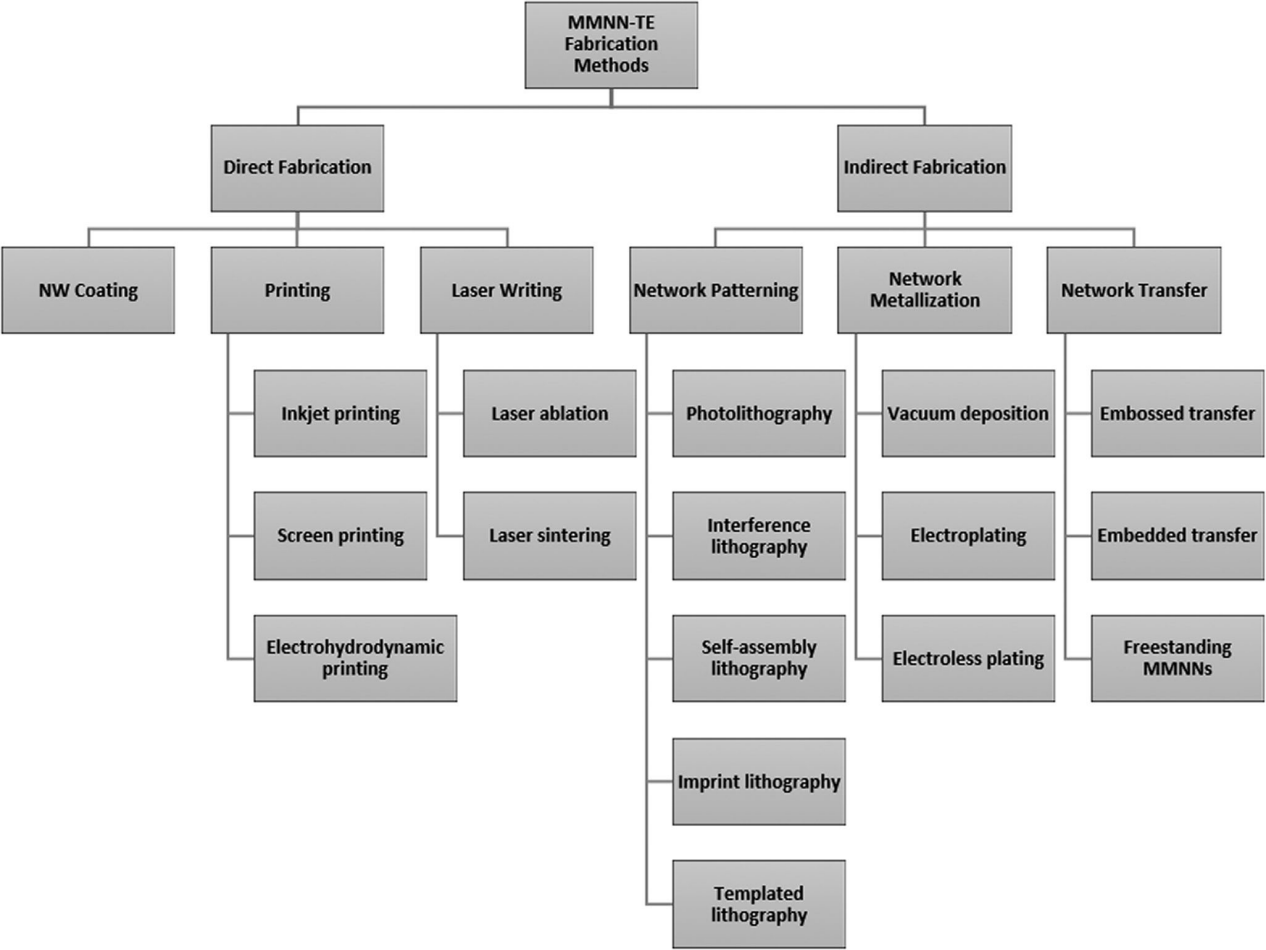
Overview of the fabrication techniques for MMNN‐based soft TEs.

This section reviews various fabrication techniques for MMNN‐based TEs that are divided into two main categories: direct fabrication methods and indirect fabrication methods, as illustrated in **Figure** **1**. The term “direct fabrication” refers to additive fabrication procedures where MMNNs are directly deposited on soft transparent substrates in a single step. The indirect fabrication process normally entails multiple steps, beginning with the creation of the network patterning on a carrier, followed by metallization of the network pattern, and ultimately the transfer of the metal network onto the desired soft transparent substrates. **Table** **1** compares the strength and weakness of various fabrication methods for MMNNs, where the resolution of vacuum deposition, electroplating, and electroless plating is not available because these metallization methods cannot directly produce micro/nanostructures but have to be combined with other lithography methods. **Table** **2** summarizes network micropatterning (lithographic) methods compared in this review.

**Table 1:** Comparison of direct fabrication methods.

	NW spin coating	NW dip coating	NW blade coating	NW rod coating	NW slot‐die coating	Inkjet printing	Electrohydrodynamic printing	Screen printing	Laser direct writing
Scalability	Medium	High	High	High	High	High	Medium	High	Low
Material waste	High	Low	High	High	Medium	Low	Low	High	Low
Pproduct consistency	Low	Low	Low	Low	Low	Medium	Medium	Medium	Medium
Cost	Low	Low	Low	Low	Medium	Medium	Medium	Medium	Medium
Environmental footprint	High	Low	High	High	Medium	Low	Low	High	Low
Technology readiness level	High	Low	High	High	High	High	Low	High	High

**Table 2:** Comparison of indirect fabrication methods.

	Patterning						Metallization		
	Photolithography	Interference lithography	Self‐assembly lithography	Imprint lithography	Templated lithography	Indeterministic lithography	Vacuum deposition	Electroplating	Electroless plating
Scalability	Medium	High	High	Medium	Medium	High	Medium	High	High
Material waste	High	High	Low	Low	Low	High	High	Low	Low
Product consistency	High	Medium	Low	High	High	Low	High	High	Medium
Cost	High	High	Low	Medium	Low	Low	High	Low	Low
Environmental footprint	High	High	Low	Low	Low	High	Low	High	High
Technology readiness level	High	Medium	Low	Medium	Low	Low	High	High	Medium

### 3.1 Direct MMNN Fabrication Approaches

Direct fabrication techniques are preferred because they directly pattern metals into micro‐ and nanostructures and are simple to use. Direct coating of metallic NW solutions is widely utilized in laboratories and industry, and MMNNs are obtained when the solvents dry out during the coating operation. Although spin‐coating is commonly used in labs due to its remarkable reproducibility, there is a significant amount of material waste. Therefore, in industries where achieving an appropriate price is challenging, R2R‐compatible processes like blade coating and slot‐die coating are favored. Printing and laser sintering processes use nanoparticle (NP) solutions. In contrast to laser sintering, which is also a cost‐effective option but has worse resolution, printing offers high resolution, great accuracy, and the capacity to fabricate objects with high aspect ratios. The laser ablation method, which has great productivity and low cost, can also be used to create micro‐patterns.

#### 3.1.1 NW Coating

Solution‐processed metal nanowire networks (MNWNs) are widely used for MMNN‐based TEs. They are typically deposited directly on the soft substrate via spin coating,^[^ ^54^ ^]^ dip coating,^[^ ^55^, ^56^ ^]^ blade coating,^[^ ^57^ ^]^ rod coating,^[^ ^58^ ^]^ and R2R compatible slot‐die coating,^[^ ^3^, ^7^, ^59^ ^]^ among other methods. Another typical scenario is the use of certain patterning techniques to create structured electrodes after the deposition of MNWNs.^[^ ^60^ ^]^ In order to prevent the precipitation of the metal nanowires (MNWs) solution, concentrations of MNWs and polymer surfactants must be kept at an appropriate level with little variation.^[^ ^3^ ^]^ The density of the coated MNWs on substrates is typically controlled by adjusting coating parameters, such as pullout velocities for dip‐coating^[^ ^56^ ^]^ and flow rates of slot‐die coating.^[^ ^7^ ^]^

**Figure 2:**
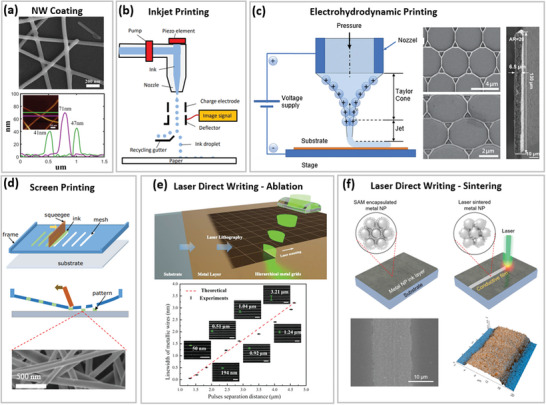
Direct MMNN fabrication approaches. a) Top‐view SEM image (top) and AFM measurement (bottom) of hydrazine (N2H4)‐treated Ag NWs. Reproduced with permission.^[^ ^54^ ^]^ Copyright 2019, Elsevier. b) Schematics showing a continuous inkjet printer. Reproduced with permission.^[^ ^67^ ^]^ Copyright 2017, MDPI. c) Left: schematic of EHD printing system setup; middle: EHD‐printed Au nanogrids; right: The cross‐sectional image of a high‐aspect ratio silver wall microstructure obtained by printing 40 layers. Left: Reproduced with permission.^[^ ^68^ ^]^ Copyright 2023, Elsevier. Middle: Reproduced with permission.^[^ ^18^ ^]^ Copyright 2016, Wiley‐VCH GmbH. Right: Reproduced with permission.^[^ ^69^ ^]^ Copyright 2021, Springer. d) Schematic of the screen printing process. Reproduced with permission.^[^ ^70^ ^]^ Copyright 2019, Nature Publishing Group. e) Top: fabrication schematic of the line‐shaped laser lithography; bottom: the relation between the linewidth of metallic wires and the pulse separat ion distance at a fixed pulse energy. Reproduced with permission.^[^ ^71^ ^]^ Copyright 2023, Wiley‐VCH GmbH. f) Schematic illustration of the metallic NP ink layer on a substrate before (top left) and after (top right) laser sintering; SEM (bottom left) and AFM (bottom right) images of the laser‐sintered Ag microstructure. Reproduced with permission.^[^ ^72^ ^]^ Copyright 2017, MDPI.

Despite the facile fabrication processes, MNWN TEs are not yet suitable for widespread deployment for a number of reasons. First, longer and thinner metal NWs help to increase optical transmittance at a lower sheet resistance;^[^ ^58^ ^]^ however, Ag NWs are typically only 10 µm long, necessitating additional effort to elongate the NWs.^[^ ^61^ ^]^ Second, in many applications like touch panels and displays where lower size dispersion is needed, the homogeneity of MNWNs is crucial.^[^ ^62^ ^]^ Therefore, polymer surfactants are necessary to improve the dispersion and stabilization of metal NW suspensions.^[^ ^63^ ^]^ However, the insulating surfactants cause charge transport barriers and deteriorate electrical conductivity. Additionally, even though polymer surfactants are employed, the shelf life is just a few months at most, which needs improvement for commercialization. Third, it is challenging to remove impurities like NPs and nanorods from the solution of synthesized NWs, which only affect optical haze and have no impact on conductance.^[^ ^62^ ^]^ Optical haze is defined as forward scattered light over total transmitted light due to the strong surface plasmon resonance (SPR) effect of noble metals. The strong scattering can cause blurriness, reduced visual appearance, and image distortion when used as the TE of displays. Fourth, a major issue with employing MNWNs as TEs is the junction resistance, which is brought on by the junction of overlapping NWs, as illustrated in **Figure** **2**a. The large surface roughness of the uneven metal NWs also makes them susceptible to creating a current shunt path when used in thin film organic devices.^[^ ^23^ ^]^ To planarize the network and get rid of the junction for better electrical conductivity, post‐treatments are needed. These include metal coating,^[^ ^58^ ^]^ annealing,^[^ ^64^ ^]^ plasmonic/electroplating/capillary‐force‐induced cold welding, chemical welding,^[^ ^54^ ^]^ and mechanical impressing.^[^ ^65^ ^]^ Moreover, the adhesion between MNWNs and substrates is typically insufficient and has to be improved for certain applications.^[^ ^66^ ^]^

#### 3.1.2 Functional Materials Printing

##### Inkjet Printing

Inkjet printing is a contact‐free direct writing method to obtain micro‐MMNNs with predesigned patterns. The ink, which is made of metallic NP suspensions, must have a low viscosity yet a high enough particle concentration (volume fraction) to enable effective printing.^[^ ^73^ ^]^ The droplets are ejected from the nozzle under pulsed pressure actuated by piezoelectricity, heat, or mechanical oscillation. Figure 2b illustrates two types of inkjet printers: the continuous inkjet printer and the drop‐on‐demand (DOD) printer. The DOD system is preferred since it only releases droplets when necessary and is hence waste‐free; in contrast, the continuous system jets ink continuously, with the discarded ink being collected by the recycling gutter. The ink's physical properties, such as density, viscosity, and surface tension, as well as the rheological dimensionless parameters derived from them, such as Weber number, Reynolds number, and Ohnesorge number, determine the entire inkjet printing process, including the formation of inkjet droplets and their impingement on the substrate.^[^ ^73^ ^]^ Therefore, to successfully complete the printing process and prevent printing inaccuracies, these parameters must be carefully optimized.

The primary issue with inkjet printing is the coffee ring effect, which occurs when solvents from droplets placed on a surface evaporate, causing the printed pattern's electrical conductivity to vary and developing a non‐uniform morphology. The term “coffee ring effect” describes a phenomenon in which the suspended particles flow toward the contact line during the evaporation process, creating a ring‐like pattern.^[^ ^74^ ^]^ Various strategies are proposed to control the morphology of inkjet‐printed materials,^[^ ^75^ ^]^ such as suppressing the coffee ring effect by weakening outward capillary flow,^[^ ^76^ ^]^ increasing inward Marangoni flow,^[^ ^77^ ^]^ and sliding the three‐phase contact line.^[^ ^78^ ^]^ However, these methods make the inkjet printing technique more difficult to use. Another problem preventing inkjet printing in soft TEs is that the printed metallic NPs often require high‐temperature sintering after printing to eliminate the ligand barriers for greater conductivity and smoother surfaces,^[^ ^43^, ^79^ ^]^ but the plastic substrates cannot withstand such a temperature.

##### Electrohydrodynamic Printing

The Electrohydrodynamic (EHD) printing system consists of a printing nozzle, a driving pump, a substrate‐moving stage, and an external high‐voltage source, as shown in Figure 2c. While the resolution of inkjet printing is primarily influenced by the nozzle size and is typically limited to about 20 µm without employing masks^[^ ^80^ ^]^ or tailored nozzles, EHD printing can reduce this limitation to a sub‐micron level^[^ ^18^, ^81^ ^]^ because the ejected droplets or jets are formed at the Taylor cone tip with the feature size significantly smaller than the nozzle under the applied electric field.^[^ ^82^ ^]^ There are different EHD printing modes, from dripping to pulsating, from jet to cone‐jet, that vary with the applied voltage and the flow rate, as well as material properties, such as ink density, viscosity, surface tension, and electrical conductivity.^[^ ^83^ ^]^ It is important to note that the frequently used electrospray^[^ ^84^ ^]^ and electrospinning^[^ ^85^ ^]^ methods are the unstable EHD dripping and jetting modes, respectively. The printed pattern is unpredictable due to the lack of control of the droplets/jets. The unstable fabrication is inappropriate for large‐area production because of the nonuniform electrical conductivity distribution resulting from the random structures. Therefore, a stable dripping or jet printing mode is essential to the realization of accurate fabrication of micro/nanostructures. The best option for printing metal structures is to use the dripping mode since metallic NP suspensions have a high NP concentration but a low ink viscosity.^[^ ^80^ ^]^ In order to produce uniform drops for high‐resolution printing, pulsed voltages are used in place of steady DC voltage, and the required microdripping mode can be achieved by controlling the voltage amplitude and duration.^[^ ^86^ ^]^ The external voltage can also be tuned to alternating voltage, which permits the nanodripping mode to contribute to MMNNs with fine feature size down to the nanoscale, as demonstrated in Figure 2c.^[^ ^18^, ^68^ ^]^ Furthermore, ultrahigh aspect ratios can be realized by utilizing a metal‐glass composite nozzle as it overcomes dislocation and collapse issues when printing multiple layers, as shown in Figure 2c.^[^ ^69^ ^]^

##### Screen Printing

Screen printing is a roll‐to‐roll compatible fabrication method featuring high throughput, high resolution (down to a few micrometers in width), low cost, scalability, and a wide range of ink options.^[^ ^87^ ^]^ Key components including screen masks (stencils), squeegees, ink reservoirs, and substrates, of flatbed and rotary screen printing are depicted in Figure 2d. For screen printing, there are several inks available that are made up of fillers, binders, and solvents, among which aqueous inks are favored over organic ones since they are more environmentally friendly.^[^ ^88^ ^]^ Ag NW inks are extensively used for fabricating soft TEs by screen printing. Comprehensive consideration must be given to ink selection, printing parameter optimization, and post‐treatment in order to balance the conductivity, transparency, and flexibility of the MNWN‐based TE.^[^ ^70^ ^]^ Mechanical pressure, thermal annealing, and plasma treatment are effective post‐treatment methods that can help printed MNWNs adhere better to soft substrates and have reduced sheet resistance.^[^ ^89^ ^]^ The as‐obtained large‐area high‐performance Ag NW‐based soft TEs are extensively used in soft transparent electronics, such as electroluminescence displays,^[^ ^90^, ^91^ ^]^ strain sensors,^[^ ^92^ ^]^ touchscreens,^[^ ^93^ ^]^ radio frequency 5G antenna,^[^ ^94^ ^]^ etc.

#### 3.1.3 Laser Direct Writing

LDW technique can be concisely categorized into laser ablation and laser sintering, as displayed in Figure 2e and Figure 2f, respectively. Laser ablation uses the light‐heat conversion process to directly write the designed MMNNs by melting or even vaporizing metal materials with thicknesses ranging from nanoscale^[^ ^95^ ^]^ to microscale.^[^ ^96^ ^]^ The larger the laser power, the thicker the metal can be patterned.^[^ ^97^ ^]^ In practice, the thickness of the MMNNs is limited by available laser power, and the high energy density required for etching thick metallic materials brings potential safety risks. Recently, metallic grids with linewidth across nanometers to micrometers are realized by using a cylindrical lens to shape the laser spot into lines.^[^ ^71^ ^]^Laser sintering consumes much smaller power density by using the localized heat source to selectively convert the metal^[^ ^98^, ^99^, ^100^ ^]^ or metal oxide^[^ ^101^ ^]^ NPs into a continuous solid state. The residual NPs after sintering are then rinsed away, leaving MMNNs on the substrate. However, the laser sintering process has strict requirements for the preparation of cluster‐free ink, careful control of the wet film thickness, and sintering parameters, etc., which limit the use of this approach. Moreover, the metal mesh fabricated by the laser‐sintering technique is prone to be porous due to the rapid evaporation of the solvent,^[^ ^102^ ^]^ which degrades the properties of the MMNNs.

Although the direct fabrication methods are convenient, it is difficult to produce MMNNs with both excellent conductivity and transparency due to low structural aspect ratios and low metal quality. While MNWNs hold an aspect ratio of 1, most other solution‐processed MMNNs have aspect ratios significantly lower than 1. So far, the highest MMNN aspect ratio of 20 is achieved by EHD printing, although the fabrication process is relatively time‐consuming.^[^ ^69^ ^]^ Other issues with solution‐processed MMNNs include poor conductivity of NP‐comprised networks due to the presence of voids and boundaries, contamination from the surfactants in precursor inks, etc.

### 3.2 Indirect MMNN Fabrication Approaches

While direct manufacturing methods create micro‐/nano‐patterns using metallic materials directly, indirect fabrication methods first create the pattern in sacrificial materials and transfer the structure to metals (metallization). Deterministic network patterning, which permits exact network design, can be accomplished via photolithography, interference lithography, self‐assembly lithography, imprint lithography, and templated lithography. However, pattern randomization is desired in some cases, such as to avoid higher‐order diffraction, and indeterministic lithography is the best way to realize it without as much effort in designing artificial patterns needed in deterministic lithography.^[^ ^103^ ^]^ The selection of metallization methods depends on the desired thickness of MMNNs and the physicochemical properties of the sacrificial material. Vapor deposition has the best control over the thickness of the deposited metal, but the deposition rate is too low to efficiently achieve metallic layers up to micrometers. Electroplating and electroless plating (ELP) are capable of fast deposition to fabricate micrometer‐thick metallic structures, but electroplating generally achieves a much higher quality.

#### 3.2.1 Network Patterning

##### Photolithography

**Figure 3:**
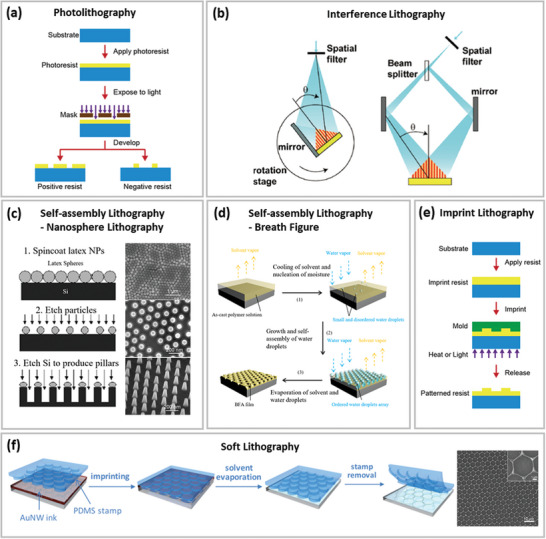
a) Schematic of photolithography processes. Reproduced with permission.^[^ ^109^ ^]^ Copyright 2018, Elsevier. b) Schematic of two‐beam interference lithography with a beam splitter (left) and with Lloyd's mirror (right). Reproduced with permission.^[^ ^109^ ^]^ Copyright 2018, Elsevier. c) Schematic of nanosphere lithography. Reproduced with permission.^[^ ^110^ ^]^ Copyright 2009, American Institute of Physics. d) Formation mechanism for BFA. Reproduced with permission.^[^ ^111^ ^]^ Copyright 2015, American Chemical Society. e) Schematic of nanoimprint lithography. Reproduced with permission.^[^ ^109^ ^]^ Copyright 2018, Elsevier. f) Schematic diagram of soft lithography. Reproduced with permission.^[^ ^40^ ^]^ Copyright 2016, American Chemical Society.

Photolithography, often known as optical lithography or UV lithography, has long been the most popular surface patterning technique commercially available. Using a photomask, it transfers geometric designs to photosensitive resist on the substrate. When the resist is exposed to UV light, it undergoes a chemical change that, depending on whether it is positive resist or negative resist, is either washed off or maintained after development. The photolithography process is schematically shown in **Figure** **3**a. Typically, photolithography is used to create patterns with micron‐sized features, but with phase‐shift masks, the resolution can be improved to a few hundred nanometers.^[^ ^104^, ^105^, ^106^ ^]^ Photolithography can make high aspect ratio patterns,^[^ ^107^, ^108^ ^]^ which can then be metalized to create thick, narrow MMNN soft TEs with excellent electrical conductivity and optical transparency.

##### Interference Lithography

Interference lithography is a mask‐free photolithography method that generates periodic nanostructures to be recorded by the photoresist. The periodic nanostructure is the spatial interference pattern created by coherent light waves, embodying the periodically distributed intensity maxima and minima. Figure 3b shows two commonly used interference lithography configurations. In Lloyd's mirror interference lithography setup, the mirror reflects half of the light beam, causing the other half to overlap and generate interference patterns. A mechanically strong connection between the two interfering beams eliminates vibrations, enabling well‐defined patterns even after a long exposure time. The configuration, however, restricts the processing area and limits large‐area fabrication. In contrast, two‐beam interference lithography can be used to develop nanostructures that are wafer‐sized.^[^ ^112^ ^]^ Typically, interference patterns are produced when two coherent beams produced by a narrow‐linewidth laser source overlap on a surface. Since the two beams are adjusted separately, vibrations are a big concern in the two‐beam configuration. This problem can be solved by employing a feedback element, like in the phase‐locked interference lithography system, and large‐area nanostructures with an aspect ratio greater than 6 are attainable.^[^ ^113^ ^]^

##### Self‐Assembly Lithography

Self‐assembly of nanostructures is a phenomenon wherein atoms, molecules, or nanoscale building blocks naturally assemble into ordered structures or arrangements with nanoscale details without any human involvement. It is one of the most practical, affordable, and high‐throughput methods for nanofabrication. Self‐assembly lithography includes the self‐assembly of solid nanospheres used in nanosphere lithography (NSL) and of liquid water droplets used in the breath figure (BF) method, which typically creates a periodic hexagonal honeycomb pattern.

NSL is a facile solution‐processed nanofabrication technique using self‐assembled hexagonally packed 2D NP arrays as masks for etching^[^ ^110^ ^]^ or deposition.^[^ ^114^ ^]^ The size of the NPs determines the self‐assembled mask's feature size, but etching the NPs can further modify the linewidth, as shown in Figure 3c. NSL offers considerable potential in terms of low cost and simple implementation, but the surface coverage with tightly packed nanospheres is modest.^[^ ^115^ ^]^ This limitation prevents NSL from large‐area fabrication and a variety of applications.

BF is named after the phenomenon of fog formation when water vapor condenses on a cold window if one breathes on it.^[^ ^111^ ^]^ Polymer films with BF‐induced hexagonally packed pores are called breath figure array (BFA) films, as shown in Figure 3d. A wide range of polymeric and non‐polymeric materials have been used for the construction of BFAs since their discovery in 1994.^[^ ^116^ ^]^ The desired materials for producing BFA films feature the capabilities of 1) forming continuous film and 2) effectively stabilizing water droplets. A majority of polymers can be used for BF patterning because they can form films if dissolved in organic solvents and they can stabilize water droplets as they are immiscible in water. With the development of the BF technique, non‐polymeric materials for BFA assembly became accessible as well, including gold NPs,^[^ ^117^ ^]^ small organic molecules,^[^ ^118^ ^]^ carbon nanotubes,^[^ ^119^ ^]^ and graphene oxide.^[^ ^120^ ^]^ Morphological control, including the pore diameter and spacing between pores of the BFA films, can be realized by adjusting parameters like solvent materials,^[^ ^121^, ^122^ ^]^ substrate materials,^[^ ^123^ ^]^ and substrate temperature,^[^ ^124^ ^]^ etc., it is possible to regulate the morphology of the BFA films, including the pore width and spacing between pores. This makes it possible to create BFAs with features as small as a few microns or even sub‐micron.

##### Imprint Lithography

Imprint lithography (IL) is a time‐saving patterning technique that makes use of an existing mold, as opposed to other methods that need several stages to generate structures from nothing. IL is typically divided into two categories: thermal IL and UV IL. Thermal IL impresses thermoplastic polymers into the imprint mold, when the polymer becomes softened at the temperature above its glass‐transition temperature. Following cooling, the polymer that has become solidified replicates the mold's complementary structures. Due to the fact that the patterned polymer may directly serve as the soft substrate, thermal IL is inherently compatible with the fabrication of numerous soft transparent devices. It is notable that recent results show that it is possible to pattern crystalline materials by thermal IL even in the absence of a glass‐transition temperature, such as hybrid perovskites.^[^ ^125^, ^126^, ^127^ ^]^ Similarly, significant work has been done to accomplish in situ metallization of microstructures embedded in imprinted polymers, through the use of the thermal IL process.^[^ ^128^ ^]^ UV IL transfers patterns to UV‐curable resists by impressing the mold into liquid resist and hardening the resist via UV exposure. Figure 3e schematically illustrates the fabrication process of UV IL.

In addition to traditional thermal and UV IL, soft lithography utilizing elastomeric stamps has been developed and has proved essential in patterning materials subjected to solution processing. Elastomeric materials, especially polydimethylsiloxane (PDMS), are used as imprint stamps in this technique. For instance, Au NWs ink is spread on the substrate and directly patterned into nano‐meshes after the solvent has evaporated via the permeable PDMS stamp,^[^ ^40^ ^]^ as shown in Figure 3f.

##### Templated Lithography

**Figure 4:**
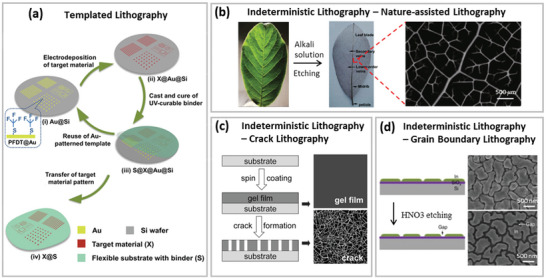
a) Schematic illustration of the templated lithography for fabricating Cu MMNNs. Reproduced with permission.^[^ ^129^ ^]^ Copyright 2023, Wiley‐VCH GmbH. b) A fresh leaf (left) is etched to dry leaf venation network (middle); right: an SEM image of the network. Reproduced with permission.^[^ ^130^ ^]^ Copyright 2020, Springer. c) Schematic of crack lithography (left) and optical microscope images of the gel film and dry film with cracks (right). Reproduced with permission.^[^ ^131^ ^]^ Copyright 2014, Wiley‐VCH GmbH. d) Schematic of grain boundary lithography (right) and SEM images of resulting structures (right). Reproduced with permission.^[^ ^132^ ^]^ Copyright 2014, Nature Publishing Group.

The template‐based method is suggested as a cost‐effective approach for reducing the “patterning‐metallization‐transfer” fabrication cycle, where a metallization template can operate for a number of cycles without noticeable deterioration. For instance, **Figure** **4**a demonstrates the schematic for fabricating the MMNNs‐based TEs using a reusable template. The SU‐8 template, which was made on rigid ITO glass and used to confine the electroplated metal meshes, can be reused for electroplating after the metal mesh has been transferred to soft substrates. Reusable templates' cycle lifespan is determined by the template's material and fabrication circumstances. For instance, SU‐8^[^ ^25^ ^]^ and SiO_2_ templates^[^ ^26^ ^]^ can provide more than 20 fabrication cycles without obvious deterioration, and Au‐on‐Si templates^[^ ^129^ ^]^ can be reused over 100 times.

##### Indeterministic Lithography

The patterning techniques discussed above produce deterministic MMNNs. Other patterning techniques that produce more random and indeterministic have also been developed for certain approaches. The easiest and most convenient way to obtain indeterministic lithographic masks is to employ objects found in nature.^[^ ^133^, ^134^ ^]^ For instance, micro‐fractals and nano‐networks can be derived from leaf venation and spiders’ silk webs for efficient solar cells and touchscreens, respectively.^[^ ^130^, ^135^ ^]^ The hierarchical venation system is chemically extracted and employed as masks for metal deposition, as shown in Figure 4b. The device's optoelectronic performance and mechanical flexibility are acceptable, despite the process's poor fabrication scalability, which is limited by the size of the natural network structures.

Another method of creating indeterministic patterns is known as “crack lithography,” which makes use of self‐cracking materials including TiO_2_ gel films,^[^ ^136^ ^]^ acrylic resin NP films,^[^ ^137^ ^]^ egg‐white,^[^ ^138^ ^]^ and Si_3_N_4_ thin films,^[^ ^139^ ^]^ among others. Different mechanisms can cause cracks to appear. For instance, as illustrated in Figure 4c, cracks coming from solution‐processed films are created during the solvent's drying process, and the width may be effectively controlled by varying the drop‐coated wet suspension film thickness.^[^ ^93^ ^]^ Another trigger for cracks can be the high stress in the deposited film such as Si_3_N_4_ thin films; to change the crack width, the substrate material is etched below the Si_3_N_4_ mask.^[^ ^139^ ^]^

Similar to crack lithography, grain boundary lithography uses the natural formation of crystal grains as masks to create MMNN patterns, but it has more control over feature size. For instance, the irregular grain boundaries of vacuum‐deposited Indium (In) thin layers are shown in Figure 4d, where the average mesh size is proportional to the thickness of the Indium film.^[^ ^132^ ^]^ By adjusting the HNO_3_ etching time, the distance between indium islands can be changed. MMNN patterns can also be generated using the grain boundaries of frozen solutions, and the size of the ice crystals' grains can be adjusted by changing the solution's concentration or by annealing for recrystallization.^[^ ^140^ ^]^

Although these indeterministic lithographic techniques are appealing options for generating MMNN patterns, the irregular patterns lead to electrical and optical nonuniformity.^[^ ^141^ ^]^ The performance of random MMNNs is found by numerical simulation to be inferior to that of ordered structures.^[^ ^142^ ^]^ Regular MMNNs not only exhibit lower sheet resistance under lower metal coverage, resulting in better optical transparency over electrical conductivity than the random pattern, but they also show a smaller internal temperature difference (defined as the difference between the maximum temperature and the network's average temperature), which results in a more uniform thermal dissipation. Due to these characteristics, ordered MMNNs perform better for soft TEs and heaters than their random‐structured counterparts.

#### 3.2.2 Network Metallization Methods

After the micro/nanostructure patterns are created, metallization takes place using several methods. Since MMNNs without pinholes are typically desired for soft TEs because of higher electrical conductivity, metallization processes should be carefully chosen to maximize cost‐effectiveness while satisfying the quality demand. Furthermore, a smooth surface is necessary for applications involving thin film deposition on top of the electrode, such as organic optoelectronic devices, because surface protrusions can penetrate the thin films and cause malfunction.

##### Vacuum Deposition

**Figure 5:**
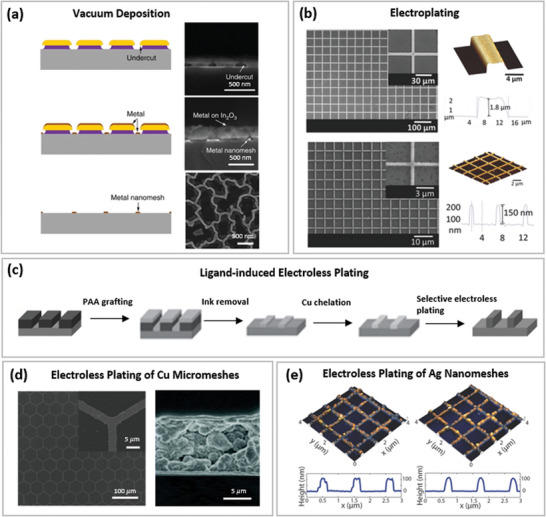
a) Schematic (left) and SEM images (right) of vacuum deposition of metal nano‐meshes through masks with the undercut. Reproduced with permission.^[^ ^132^ ^]^ Copyright 2014, Nature Publishing Group. b) SEM (left) and AFM (right) images of electroplated copper mesh on the FTO glass substrate with microstructures (top) and nanostructures (bottom). Reproduced with permission.^[^ ^6^ ^]^ Copyright 2016, Wiley‐VCH GmbH. c) Localized LIEP process for metal deposition. Reproduced with permission.^[^ ^149^ ^]^ Copyright 2011, Wiley‐VCH GmbH. d) SEM characterization of Cu meshes catalyzed by Pd NPs (left) and Cu flake microparticles (right). Left: Reproduced with permission.^[^ ^150^ ^]^ Copyright 2018, American Chemical Society. Right: Reproduced with permission.^[^ ^151^ ^]^ Copyright 2018, Royal Society of Chemistry. e) AFM images of solution‐grown Ag meshes before (left) and after (right) rapid thermal annealing. Reproduced with permission.^[^ ^152^ ^]^ Copyright 2016, Wiley‐VCH GmbH.

Vacuum deposition, which involves depositing metal materials on the patterned substrate using evaporation or sputtering under high vacuum, can be used to create MMNNs. This process is often followed by a lift‐off operation. This is often appropriate for thin‐film deposition with sub‐micron thickness. For high‐vacuum conditions, expensive equipment is needed, and the bulk of evaporated/sputtered materials are wasted. Although some research incorporates electroplating after vacuum depositing an ultra‐thin metal seed layer,^[^ ^134^ ^]^ which somewhat decreases waste, it does not fundamentally change the complexity of the process. Another disadvantage of vacuum deposition techniques is that the resist mask needs to fulfill a strict requirement: it needs to have a vertical sidewall or even an undercut for full lift‐off and to realize smooth MMNN surface. Otherwise, the deposited metal materials in and out of the structure‐defined area could link, preventing the lift‐off process from working or leaving the MMNNs with “rabbit ears”^[^ ^143^ ^]^ that might cause thin‐layer devices to short circuit. **Figure** **5**a illustrates how well‐formed deposited MMNNs can be achieved using deposition masks with undercuts, however doing so also makes fabrication more challenging.^[^ ^132^ ^]^

##### Electroplating

Electroplating is a low‐cost, vacuum‐free, high‐throughput, and mature fabrication method for metallization, capable of depositing a‐few‐microns‐thick metals within several minutes, making freestanding MMNNs possible.^[^ ^144^ ^]^ Metal is deposited by electroplating when metal ions in the plating solution go to the cathode and are transformed into metal atoms, which then build up on the cathode. Metalized micro/nanostructures via electroplating are confined by patterned insulating masks on conductive supports, and the electroplated MMNNs are dense and pinhole‐free, devoid of percolation or large junction resistance, as shown in Figure 5b. The mask materials include photoresists,^[^ ^6^ ^]^ fluoropolymers,^[^ ^145^ ^]^ sputtered SiO_2_,^[^ ^26^ ^]^ and other materials, which are selected based on compatibility with the entire fabrication process. The MMNNs are detachable from the conductive support, and they can be transferred to various soft substrates or used as freestanding electrodes.^[^ ^146^, ^147^, ^148^ ^]^

##### Electroless Plating

ELP, also known as chemical plating or autocatalytic plating, achieves metal deposition by the chemical reduction of metal cations through autocatalysis in a liquid bath. The first stage in ELP is to plant the catalytic seeds, for which numerous strategies have been developed. For instance, the ligand‐induced ELP, which was developed a decade ago, is based on the covalent grafting of a thin layer of poly‐acrylic acid (PAA), which serves as the ligand to complex copper ions and eventually chemically reduces the ions into Cu^0^ species as a seed layer for the electroless copper growth.^[^ ^153^ ^]^ MMNNs can be produced by combining lithographic patterning with ligand‐induced ELP,^[^ ^149^ ^]^ as illustrated in Figure 5c. To skip the reduction of copper ions, oleate ligand‐capped Cu flakes can be employed and serve as nucleation sites for Cu plating.^[^ ^151^ ^]^ Apart from Cu, Pd can also serve as the catalyst to initiate the ELP process of Cu, where the use of poly‐dopamine interlayer can significantly improve the adherence of plated MMNNs to substrates.^[^ ^128^ ^]^

It is worth noting that the ELP material system and fabrication techniques can significantly influence the quality of the plated metal. Figure 5d compares Cu MNs catalyzed by Pd NPs and Cu flakes: while Pd NP catalysts contribute to compact and smooth Cu coating,^[^ ^150^ ^]^ the oleate‐ligand‐catalyzed Cu MN consists of Cu flake microparticles with obvious pinholes.^[^ ^151^ ^]^ Besides Cu, Ag is also widely adopted for ELP, utilizing the Tollens’ reaction, but the plated Ag needs rapid thermal annealing to reduce surface roughness, as shown in Figure 5e.^[^ ^152^, ^154^ ^]^

### 3.3 Network Transfer

MMNN‐based TEs are often first developed on rigid substrates since the majority of lithographic methods were created for the semiconductor industry and are compatible with chip fabrication. They need to be transferred to soft substrates. Afterwards, several techniques including surface contacting, UV imprinting, and thermal imprinting, can be used to accomplish MMNN transfer. According to whether the transferred MMNNs protrude out from the substrate or are completely embedded in the substrate, we refer to these transfer methods as “embossed transfer” and “embedded transfer”. MMNNs with a thickness of only a few microns can, in exceptional cases, be peeled off the substrate and used as a freestanding optoelectronic component.

#### 3.3.1 Embossed Transfer

**Figure 6:**
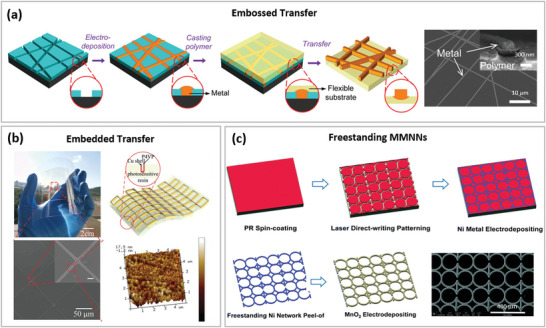
a) Schematic illustration and SEM characterization of MMNN soft TEs manufactured by embossed transfer. Reproduced with permission.^[^ ^26^ ^]^ Copyright 2019, Wiley‐VCH GmbH. b) Digital photo, SEM images, AFM image, and schematic of the embedded soft TE. Reproduced with permission.^[^ ^163^ ^]^ Copyright 2023, Wiley‐VCH GmbH. c) The fabrication schematic and SEM characterization of the freestanding MnO_2_@Ni network supercapacitor electrode. Reproduced with permission.^[^ ^148^ ^]^ Copyright 2017, Royal Society of Chemistry.

Embossed transfer leaves transferred MMNNs partially or completely protrude from the target substrate surface. Water‐soluble polymer polyvinyl alcohol (PVA) is a preferred carrier to complement the transfer process with simple “wrapping‐transfer‐release” steps.^[^ ^155^ ^]^ By applying a small amount of pressure, structured MMNN soft TEs that have been deposited on a PDMS mold can be transferred directly to the functional layer, such as a PEDOT:PSS layer.^[^ ^23^ ^]^ An alternate transfer method that increases adhesion between the transferred MMNNs and the soft substrate is the use of an adhesive bonding layer, such as the UV‐curable resist.^[^ ^156^, ^157^, ^158^ ^]^ A remarkable achievement is shown in **Figure** **6**a, which demonstrates the transfer of MMNNs grown in the trenches of SU‐8 microstructures to PDMS/plastic substrates. The metal “cap” that extends beyond the SU‐8 trenches is captured by the PDMS/plastic films and used to overcome the adhesion between the MMNN and the confinement, allowing the transfer to occur.^[^ ^25^ ^]^

A single layer of PEDOT:PSS can typically planarize MMNNs with a thickness of less than 40 nm standing on the surface of the substrate, but a thicker metal layer can result in a short circuit in multi‐layer thin‐film devices like OLEDs.^[^ ^159^ ^]^ On the other hand, decreasing the thickness of MMNNs results in much decreased electrical conductivity, which has a negative impact on the functionality of the devices. Thin MMNNs can be combined with other conducting materials, such as PEDOT:PSS (PH1000) and graphene^[^ ^160^, ^161^, ^162^ ^]^ to provide greater conductance, however, this complicates production, reduces optical transmittance, and limits the use of soft TEs. Therefore, it is preferable to completely embed the MMNNs into the substrate to address issues with rough surfaces and poor adhesion without compromising the soft TEs' optoelectronic capabilities.

#### 3.3.2 Embedded Transfer

Embedded transfer happens when the entire transferred MMNN is embedded into the soft substrate, leaving a smooth surface after transfer, as shown in Figure 6b.^[^ ^163^ ^]^ In many applications, the embedded transfer is desirable, particularly in multi‐layer thin‐film devices where the MMNN protrusion typically impales the deposited thin layers. The MMNNs can typically be completely embedded into soft substrates using a UV imprint or thermal imprint process.^[^ ^6^, ^69^, ^158^, ^164^, ^165^ ^]^ Additionally, the soft TE with completely embedded MMNN exhibits excellent flexibility and durability against mechanical deformation due to the MMNN being embedded in the substrate from three sides.^[^ ^161^ ^]^

#### 3.3.3 Freestanding MMNNs

The micron‐thick MMNN can be peeled off and used as an independent component in addition to being transferred to soft substrates to develop ultra‐thin and ultra‐soft devices.^[^ ^147^, ^148^ ^]^ An ultrathin freestanding Ni mesh with exceptional optoelectronic performance (conductivity = 3×10^4^ S cm^−1^, transparency > 84% transmittance), excellent mechanical flexibility, and stability is employed as a high‐performance supercapacitor as shown in Figure 6c.^[^ ^148^ ^]^

## 4 Performance Evaluation

Since the properties of TEs have a significant impact on the entire performance of electronic devices, it makes sense to first determine the criteria for selecting soft TEs. When designing soft TEs, numerous evaluation indexes must be taken into consideration. The first set of qualities is the optoelectronic ones, such as electrical conductivity, optical transparency, and haze. Second, the mechanical stability with regard to softness, surface smoothness, and adhesion. The environmental stability against heat and chemicals is the third factor. As a result, this section will cover the soft TEs' performances as well as the elements that affect them.

### 4.1 Optoelectronic Properties

The primary purposes of soft TEs are to transmit light and conduct electricity. Therefore, optical transparency and electrical conductivity are two important performance metrics that characterize the quality of soft TEs. Higher electrical conductivity frequently implies reduced optical transparency for MMNNs. The simultaneous improvement of both is challenging. To compare performance and improve the design of soft TEs, many researchers are striving to analyze the trade‐off between electrical and optical characteristics.

#### 4.1.1 Electrical Performance

MMNN‐based TEs showed exceptionally high electrical conductivity, which is particularly exciting for large‐area optoelectronic applications.^[^ ^25^ ^]^ The distribution of the metallic network on the substrates, its geometric properties, and the materials it is comprised of all have a significant impact on the sheet resistance of MMNN‐based electrodes. Individual metal wires' geometric parameters, which are influenced by the process of production, include their width, length, and size distribution. The most significant factor is the density of the metal, since decreasing the sheet resistance is achieved by raising the density of metal wires over the percolation threshold. However, the resistance's lowering tendency will pause when the density of metal wires is sufficient. By employing high‐conductivity metals, improving the aspect ratio of metal wires, and creating new metal wire shapes, the electrical conductivity of the MMNN‐based TEs can be further improved.^[^ ^166^ ^]^

#### 4.1.2 Optical Performance

Along with electrical conductivity, optical transparency must also be taken into account. This is often done by measuring the film's light transmittance at 550 nm. The majority of MMNNs maintain an optical transmittance that is essentially unchanged in the visible range and is superior to ITO in the IR range. As a result, MMNNs have a great potential in IR applications that demand high transparency.^[^ ^32^ ^]^ The line width and spacing of the metal lines on the substrates have an impact on optical transmittance. Because fewer photons scatter in metal networks with narrower lines, they exhibit better transmittance. The spatial density of conducting metal in the MMNNs is therefore inversely related to optical transmittance.^[^ ^167^ ^]^

The transmittance is not the only relevant component to consider when measuring considerable light scattering; the haze factor is also important.^[^ ^168^ ^]^ The ratio of diffuse to direct light transmission is known as the haze factor. Each application's specific requirements for industrial applications are different. For example, the haze factor must be less than 2% for display applications, whereas greater values are needed for solar cells to increase photovoltaic efficiency. Therefore, it is difficult for the MMNNs to meet the industrial requirements without being able to control the haze factor.^[^ ^169^ ^]^ Numerous research has demonstrated that the metallic network's size and spatial distribution can be used to adjust the haze factor.^[^ ^170^ ^]^ However, more research should be done on this subject to further develop a metric for evaluating the haze factor of MMNN TEs.

#### 4.1.3 Figure of Merit

**Figure 7:**
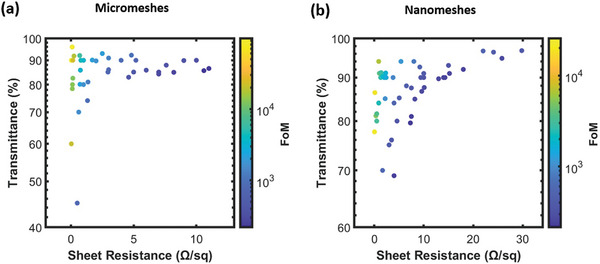
Optoelectronic properties of various soft TEs reported in recent literature based on a) micro‐meshes and b) nano‐meshes.

High optical transparency normally makes aesthetic sense, but it is essential for applications involving light interactions, which mostly rely on soft TEs. Therefore, the creation of high‐performance soft TEs is essential to keep up with the rapid development of electronic technology. High‐performance soft TEs are often defined by their mechanical flexibility, electrical conductivity, optical transparency, long‐term stability, manufacturing cost, and scalability. Although the majority of them are independent of one another, the electrical and optical characteristics of soft TEs are inextricably linked. Therefore, it is necessary to evaluate soft TEs' electrical and optical performance jointly and to compare it to recognized benchmarks. The fundamental goal is to identify a parameter that can show how the optical transmittance of a film and its sheet resistance relate to one another. The most used metric for rating optoelectronic properties is the figure of merit (FoM). In order to calculate FoM, which is the ratio of electrical conductivity to optical conductivity, the following frequently utilized equation is used.^[^ ^171^, ^172^ ^]^

(1) $$FoM=\frac{\sigma_{dc}}{\sigma_{opt}}=\frac{188.5}{R_{sheet}\left(\frac{1}{\sqrt{T_{550nm}}}-1\right)}$$

where σ_*dc*_ is the electrical conductance, σ_*opt*_ is the optical conductance, *R_sheet_* is the sheet resistance, and *T*_550*nm*_ is the optical transmittance at a wavelength of 550 nm. **Tables** **3** and **4** and **Figure** **7** summarize the optoelectronic performance of MMNN TEs that have recently been published in the literature. Even though the value of FoM aids in assessing the trade‐off between optical transparency and electrical conductivity, it lacks physical models and is still unable to explain the connection between the features of MMNNs and their optoelectronic attributes. Moreover, the effect of light scattering is neglected while calculating FoM.

**Table 3:** Optoelectronic properties of various metal micro‐mesh‐based TEs reported in recent literature.

Rs [Ω □^−1^]	T [%]	FoM	Application	Reference
0.04	90	9.7×10^4^	Solar cells and heaters	[25]
0.1	96	9.1×10^4^	Heaters	[151]
0.15	90	2.3×10^4^	Heaters and EMI shielding	[69]
0.03	60	2.2×10^4^	Electrodes	[138]
0.24	92	1.8×10^4^	EMI shielding	[144]
0.11	78	1.4×10^4^	Sensors and heaters	[173]
0.12	80	1.3×10^4^	Electroluminescent devices	[174]
0.15	83	1.2×10^4^	Thermotherapeutic skin patches	[175]
0.7	92	6.4×10^3^	Supercapacitors	[176]
0.79	90	4.4×10^3^	TSPs	[177]
1	90	3.5×10^3^	Heaters	[6]
0.75	86	3.2×10^3^	Electrodes	[165]
0.72	80	2.2×10^3^	Sensors	[9]
1.7	90	2.0×10^3^	Heaters	[178]
2.5	93	2.0×10^3^	Electroluminescent devices	[163]
2	90	1.7×10^3^	Supercapacitors	[179]
1	80	1.6×10^3^	Electroluminescent devices	[150]
0.62	70	1.6×10^3^	Supercapacitors	[180]
3	91	1.3×10^3^	OLEDs	[85]
4.82	92	9.5×10^2^	Electrodes	[51]
1.32	74	8.8×10^2^	Dye‐sensitized solar cells	[181]
4	90	8.7×10^2^	OLEDs	[158]
3	86	8.0×10^2^	Solar cells	[161]
0.5	45	7.7×10^2^	Electrodes	[131]
2.95	85	7.5×10^2^	Sensors	[47]
5.18	90	6.7×10^2^	Photodetectors	[182]
9.1	99	4.6×10^2^	Heaters	[41]
5	85	4.5×10^2^	TSPs	[97]
4.6	83	4.2×10^2^	Electrodes	[71]
6	86	3.9×10^2^	Sensors	[82]
9	90	3.8×10^2^	Polymer solar cells	[154]
7.6	88	3.7×10^2^	Supercapacitors	[183]
10	90	3.5×10^2^	Heaters and TSPs	[93]
7	85	3.2×10^2^	OLEDs	[184]
7	84	3.0×10^2^	Electrodes	[185]
8.2	85	2.7×10^2^	Wearable electronics	[186]
11	87	2.3×10^2^	Heaters	[187]
10.6	86	2.2×10^2^	Heaters and TSPs	[147]

**Table 4:** Optoelectronic properties of various metal nano‐mesh‐based TEs reported in recent literature.

Rs [Ω □^−1^]	T [%]	FoM	Application	Reference
0.06	78	2.5×10^4^	OLED, heaters, and TSPs	[129]
0.13	86	1.9×10^4^	Electrodes	[145]
0.9	94	6.7×10^3^	Sensors	[188]
0.34	81	5.0×10^3^	EMI shielding	[189]
1.03	91	3.8×10^3^	Sensors	[190]
0.5	82	3.5×10^3^	Sensors	[35]
0.5	80	3.2×10^3^	Solar cells	[164]
1.4	91	2.9×10^3^	Hyperthermia patches	[191]
1.3	90	2.7×10^3^	Smart contact lenses	[192]
0.9	84	2.3×10^3^	Electroluminescent devices	[26]
1.7	90	2.1×10^3^	Supercapacitors	[10]
2	90	1.7×10^3^	Conductive paper	[193]
2.32	91	1.7×10^3^	Sensors and heaters	[194]
2.4	90	1.5×10^3^	Electrodes	[137]
5.4	90	1.1×10^3^	Solar cells	[195]
2.2	85	1.0×10^3^	Electrodes	[135]
3.8	90	9.2×10^2^	Heaters	[196]
8	94	7.5×10^2^	Electrodes	[18]
1.68	70	5.7×10^2^	Electrodes	[197]
9.2	93	5.2×10^2^	Electrodes	[94]
4	84	5.2×10^2^	LEDs and sensors	[198]
22	97	5.1×10^2^	Electrodes	[56]
5.05	85	4.5×10^2^	Sensors and heaters	[199]
6.5	88	4.4×10^2^	Solar cells	[200]
24.1	96	4.2×10^2^	Electrodes	[63]
3	75	4.1×10^2^	Neural electronics	[201]
10	91	3.9×10^2^	PLEDs	[202]
29.8	97	3.9×10^2^	Electrodes	[203]
9.4	90	3.7×10^2^	Heaters	[204]
3.5	76	3.7×10^2^	Electrodes	[152]
7.5	88	3.6×10^2^	Electrodes	[134]
10	90	3.5×10^2^	Electrodes	[205]
4.6	80	3.5×10^2^	Sensors	[38]
15	93	3.4×10^2^	Electrodes	[196]
10.1	88	2.7×10^2^	Sensors	[206]
25.8	95	2.7×10^2^	Electronic skins	[14]
8.2	85	2.7×10^2^	Epidermal electronics	[207]
9.6	87	2.7×10^2^	Sensors	[208]
13	90	2.6×10^2^	Heaters and electrodes	[33]
12.5	89	2.6×10^2^	Heaters	[209]
14	90	2.5×10^2^	Electrodes	[210]
18	92	2.5×10^2^	Triboelectric nanogenerators	[54]
14.29	90	2.4×10^2^	Polymer solar cells	[57]
4	69	2.3×10^2^	Epidermal electronics	[90]
7.5	81	2.3×10^2^	Organic solar cells	[156]
7.3	80	2.1×10^2^	Electroluminescent devices	[91]

### 4.2 Mechanical Properties

Mechanical stability is another crucial aspect of soft electronics, which is significantly influenced by the mechanical characteristics of soft TEs. The softness, surface roughness, and metal‐substrate adhesion are all part of the mechanical characteristics.

#### 4.2.1 Softness

As a crucial part of soft electronic devices, soft TEs must be extremely flexible, exhibit little change in characteristics when mechanically deformed, and be compliant to apply to various surfaces. The greatest amount of bending that soft TEs can tolerate before losing a significant amount of their conductivity is used to measure their flexibility. MMNN‐based TEs are superior to conventional ITO electrodes because they are more flexible, foldable, and stretchable.^[^ ^193^ ^]^ MMNN‐based TEs have minimal conductivity loss after repetitive bending, under which conventional ITO‐based TEs exhibit significant conductivity loss.^[^ ^28^, ^193^ ^]^ Stretchability, in addition to flexibility, is crucial in the development of soft TEs for wearable electronics. Stretchability of soft TEs is evaluated using the maximum tensile strain that the electrodes can withstand without significantly losing their conductivity. Silver nanowire‐based soft TEs showed reasonable stretchability up to 20% strain with no apparent conductivity changes.^[^ ^210^ ^]^ Silver nanowire/polymer composite films were used to create soft TEs that could endure up to 71% bending or stretching strain.^[^ ^211^ ^]^ Stretchable metal‐mesh TEs can effectively sustain more than 50% strain while maintaining excellent optoelectronic properties.^[^ ^212^ ^]^ A highly stretchable TE made of gold nano‐mesh slightly lost its conductivity at a strain of about 150%.^[^ ^132^ ^]^ A highly stretchable MMNN TEs with remarkable optoelectronic performance and only 30% of conductivity loss under a 70% tensile strain was also developed for wearable electronics.^[^ ^196^ ^]^

The flexibility and stretchability of MMNN‐based electrodes, despite the inherent ductility of materials like Au, Ag, Cu, and Ni, heavily rely on the rational structural design of the network. In the case of metallic NW networks, the deformation of nanowires occurs through correlated tensile fractures, compression‐induced kinking, and bending resulting from the strain in the network.^[^ ^213^ ^]^ To achieve improved strain tolerance and fatigue performance, buckled NW structures are generated by pre‐stretching the elastomer substrate.^[^ ^214^, ^215^ ^]^ Additionally, the curvature of deposited NWS can be tailored through different dispensing approaches. While spin‐coated nanowires tend to straighten during the spin‐coating process,^[^ ^200^ ^]^ sprayed nanowires and electrospun nanofibers exhibit a curly state after coating, demonstrating excellent reliability against mechanical bending and stretching.^[^ ^190^ ^]^ This concept applies to other MMNNs as well, where a deliberate wavy micro/nanostructure design plays a crucial role in enhancing the flexibility and stretchability of the soft electrode. For example, serpentine and zigzag meshes have proven to sustain larger stretching compared to square and hexagon meshes. Further optimization of the MMNN structure is likely to lead to even better mechanical performance.^[^ ^212^ ^]^

#### 4.2.2 Mechanical Adhesion

In addition to softness, MMNN‐substrate adhesion is a critical issue in the use of MMNN‐based TEs. The poor adherence of the metal to the substrates, in addition to their roughness and small effective electrical area, limits the broad use of MMNN‐based TEs in thin film electronic devices and degrades the devices' mechanical and electrical stability. The best technique to increase the adherence of MMNNs to soft polymeric substrates is to embed and mechanically anchor them.^[^ ^216^ ^]^ In addition to these mechanical techniques, a great deal of research is being done on chemical methods to improve the adhesion of the MMNNs to soft polymeric substrates. Polydopamine adhesives were used to promote the mechanical adherence of metallic silver networks to flexible PET substrates, which considerably increased the mechanical flexibility of the soft TE.^[^ ^217^ ^]^ By utilizing an Au‐S bond, the mechanical stretchability of the soft TE was greatly increased by strengthening the mechanical adhesion of gold nanonetworks with a stretchable PDMS substrate.^[^ ^218^ ^]^ Similarly, by applying 11‐aminoundecanoic acid to the substrate, the adhesion of silver nanowire networks with elastic PDMS is increased.^[^ ^219^ ^]^ A PDMS surface with densely self‐assembled amine is demonstrated to have a strong adhesion force with the silver nanowire network to improve the soft TEs' flexibility and stretchability.^[^ ^220^ ^]^ Also, the adhesion between silver nanowires and substrate was improved by using a hybrid nanomaterial made up of a network of silver nanowires and p‐type reduced graphene.^[^ ^162^ ^]^

#### 4.2.3 Surface Roughness

In order to attain sufficiently high electrical conductivity required in numerous applications, MMNNs must be deposited in thick layers on substrates. These layers can easily result in electrical short circuits, high leakage currents, and low quantum efficiency in optoelectronic devices, which prevents MMNN TEs from being compatible with thin‐film multi‐layer electronic devices. Many studies are being done to develop soft TEs with smooth surface properties that are based on MMNNs. The ideal technique to reduce the roughness of the soft TEs' surface is to directly insert MMNNs into the polymeric substrate materials.^[^ ^6^, ^216^ ^]^ The alternative idea is to bury MMNNs beneath the surface of other functional materials including transparent resin matrices,^[^ ^205^ ^]^ conductive coatings,^[^ ^180^ ^]^ and buffer layers.^[^ ^203^ ^]^

### 4.3 Stability

For MMNN‐based TEs to be used in industrial applications, environmental stability is just as important as other performance criteria. When exposed to extreme conditions, such as high temperatures and high humidity, MMNN is prone to corrosion and breakdown. Although recent research has shown that their integration in a variety of electronic devices is successful, systematic investing regarding stability is still lacking, which is essential for the long‐term integration of MMNN TEs in thin film electronic devices. Further research on stability improvement is required due to insufficient data on the aging of soft TEs in diverse conditions and the lack of effective protective layers.

#### 4.3.1 Environmental Stability

MMNNs are susceptible to air corrosion, particularly when moisture or other harmful gases are present. Humidity, light, electrical stress, and poisonous chemicals are just a few of the factors that might cause MMNNs to degrade. Reducing the area of the MMNNs exposed to the environment is one strategy to boost chemical stability because the degree of degradation starts from this exposure area. Stable materials like metal oxides, graphene materials, and highly stable metals can be deposited for this purpose. For instance, the stability of silver NW‐based TEs was improved by using protective coatings made of metal oxides including zinc oxide (ZnO) and tin oxide (SnO_2_).^[^ ^221^ ^]^ Similar to this, soft TEs made of silver nanowires network are coated with ultra‐thin films of aluminum oxide (Al_2_O_3_) to improve their endurance to heat and the environment.^[^ ^222^ ^]^ Graphene also protected the silver nanowire‐based TEs against high moisture content even at high temperatures.^[^ ^209^ ^]^ A protective coating made of hydrophobic dodecanethiol is employed to increase the stability of copper nanowire‐based TEs, which demonstrated good stability for 12 hours at 85 °C and 85% RH under humid conditions.^[^ ^223^ ^]^ The metal network was also embedded in soft polymeric substrates to increase the chemical stability of the MMNN TEs, which showed exceptional stability under high‐humidity and high‐temperature conditions (60 °C, 85% RH).^[^ ^6^ ^]^

#### 4.3.2 Operational Stability

When current runs through soft TEs constantly and for an extended length of time, as is the case for some applications like OLEDs, polymer solar cells, and transparent heaters, thermal instability may occur, which poses a major threat to the long‐term durability of these soft devices.^[^ ^224^ ^]^ High temperatures would worsen corrosion and morphological instability and the MMNN junctions are more prone to failure because they regularly demonstrate higher temperatures than the surroundings.^[^ ^225^ ^]^ Surface protection strategies have been developed by employing organic, inorganic, or carbon‐based materials to address the thermal stability issue of MMNN‐based TEs for their broad applications. For example, the AgNW‐based TEs embedded in the reinforced composite made of glass fabric can survive temperatures of up to 250 °C for 2 h.^[^ ^226^ ^]^ MMNNs are also embedded in polyimide film, where the TE shows remarkable thermal stability up to 400 °C.^[^ ^227^ ^]^ MMNN‐based TEs were protected by graphene, which also provided moisture protection, allowing them to maintain stable operation at 300 °C.^[^ ^209^ ^]^ For the protection of metallic networks, high melting‐temperature inorganic capping layers of ZnO and TiO_2_ were also utilized, and the TEs were able to endure thermal processing at 300 °C with no loss in conductivity.^[^ ^202^, ^228^ ^]^

## 5 Applications

**Figure 8:**
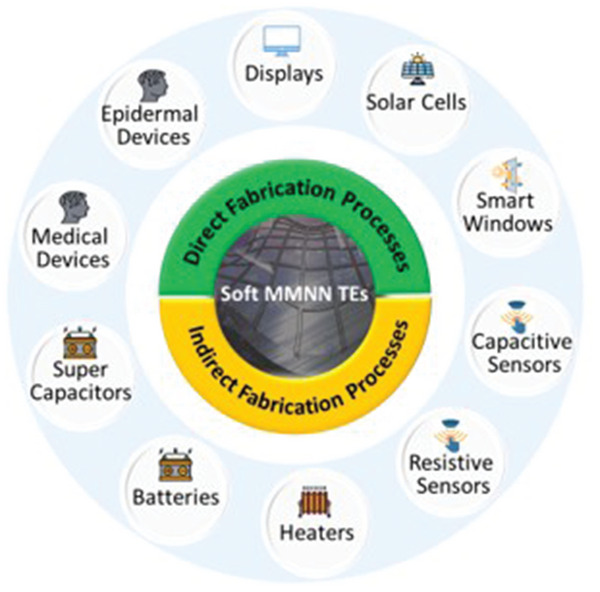
Application overview of MMNN‐based soft TEs in optoelectronics, bioelectronics, energy storage devices, tactile sensors, and heaters.

MMNN‐based TEs have higher overall performance than other soft TE types, however, what makes them so appealing and widespread is their extremely high FoM. Lightweight, invisible soft electrodes are usually advantageous for soft electronic devices, but some systems that interact with light require high‐FoM electrodes. This section outlines the broad electronic applications of MMNN‐based TEs, as illustrated in **Figure** **8**, with a focus on those where high optical transparency and high electrical conductance are required for optimal performance.

### 5.1 Optoelectronics

Optoelectronic devices are typically categorized into electroluminescent light‐emitting devices, photodetectors, and photovoltaics. Due to the more flexible device structure of photodetectors, where the active materials can be exposed to illumination without covering the electrodes, MMNN TEs are utilized in photodetectors somewhat less frequently than in other types of devices.^[^ ^229^ ^]^ However, MMNN TEs can still be advantageous for photodetectors, at least in terms of mechanical flexibility.^[^ ^182^, ^230^ ^]^

#### 5.1.1 Light‐Emitting Devices

**Figure 9:**
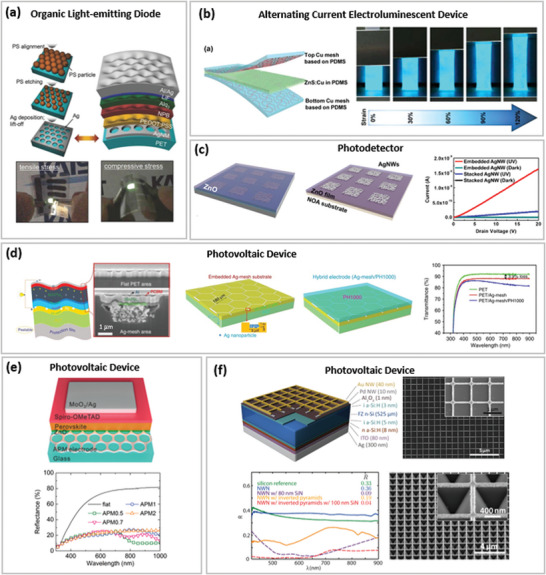
a) Flexible nanostructured OLEDs with a plasmonic Ag nano‐mesh anode. Top: schematic illustration of the Ag nano‐mesh fabrication procedure and the OLED structure; bottom: photographic images of bending tests of flexible OLEDs. Reproduced with permission.^[^ ^231^ ^]^ Copyright 2015, Wiley‐VCH GmbH. b) Schematic diagram (left) and stretching tests (right) of the ACEL device based on Cu‐MN soft TEs. Reproduced with permission.^[^ ^174^ ^]^ Copyright 2023, Wiley‐VCH GmbH. c) Device schemes of ZnO‐based UV photodetectors with embedded (left) and stacked (middle) Ag NW network electrodes and photocurrent characteristics of them (right). Reproduced with permission.^[^ ^230^ ^]^ Copyright 2020, American Chemical Society. d) Left: device architecture and cross‐section SEM image of the flexible perovskite solar cell; middle: structure illustration of the flexible Ag mesh‐embedded substrate; right: transmission spectra of different substrates. Reproduced with permission.^[^ ^161^ ^]^ Copyright 2016, Nature Publishing Group. e) Top: schematic device architecture of a perovskite solar cell with Ag nano‐mesh electrodes; bottom: reflection spectra of various electrodes. Reproduced with permission.^[^ ^233^ ^]^ Copyright 2019, Wiley‐VCH GmbH. f) Top left: device schematic of the solar cell; top right: SEM images of the NW electrode on top of the silicon half‐cell; bottom left: experimental measurements (solid lines) and simulations (dashed lines) of reflection spectra; bottom right: SEM images of a metallic NW network with integrated inverted nanopyramids. Reproduced with permission.^[^ ^234^ ^]^ Copyright 2016, American Chemical Society.

When it comes to electroluminescent devices, such as light‐emitting diodes (LEDs) LEDs and alternating current electroluminescent (ACEL) devices, the use of MMNN TEs can significantly increase device efficiency. However, to accomplish so, a good selection of metal materials for a work function match is necessary to increase the efficacy.^[^ ^184^ ^]^ The flexible organic light‐emitting diode (OLED) in **Figure** **9**a uses plasmonic Ag nano‐mesh soft TEs to increase light extraction and enhance the OLEDs' external quantum efficiency (EQE).^[^ ^231^ ^]^ The electroluminescence (EL) spectra are also engineered using the nano‐mesh electrode since the MMNN TE causes a blue shift at increased current.^[^ ^232^ ^]^ A potential profile created by the overlap of neighboring nano‐mesh's electric fields may inject current and emit light even in the uncovered area of the active region. In this case, the MMNN promotes injection of the spatially nonuniform current among the planes of the quantum wells, causing position‐dependent EL intensity and a blue shift. However, the strong light diffraction caused by the nanostructure allows the emitted light to appear angle‐dependent, hindering the use of nanostructured MMNN‐based TEs in displays. Metal micro‐mesh soft TEs with feature sizes significantly larger than the visible wavelength possess mild light diffraction and scattering,^[^ ^161^ ^]^ but the large spacing may cause non‐uniform light emission with an effective region that is only in the vicinity of the metal nano‐mesh.^[^ ^159^ ^]^ Therefore, it is essential to develop an appropriate MMNN structure for consistent and effective luminescence. Flexible and stretchable ACEL devices can be fabricated based on elastomeric PDMS substrates.^[^ ^174^, ^211^ ^]^ Figure 9b displays a Cu MN‐based ACEL device that can survive a 120% strain.

#### 5.1.2 Photodetectors

Ag NWs are widely employed as soft TEs for a range of photodetectors, including metal‐oxide heterojunction Schottky photodetectors,^[^ ^235^ ^]^ capacitive ZnS:Cu photodetectors,^[^ ^236^ ^]^ conventional ZnO photodetectors,^[^ ^237^, ^238^ ^]^ and perovskite photodetectors,^[^ ^239^ ^]^ etc. Although Ag NWs are the most often utilized material for MMNN TEs used in photodetectors, Au networks may be a better option for specific devices, such as perovskite photodetectors, taking the band alignment into consideration. High‐performance CH_3_NH_3_PbI_3_ perovskite photodetectors are realized using flexible transparent Au network electrodes with conductivity and transparency similar to commercial ITO electrodes.^[^ ^182^ ^]^ The MNWN's multifunctionality helps the device work better. First, the interfacial surface area of the active material increases with embedded metallic networks; second, the dense NW offers short paths for collecting photo‐generated carriers; and third, the NW‐induced light scattering and trapping improves light absorption.^[^ ^230^, ^240^ ^]^ This is demonstrated by comparing the performance of ZnO photodetectors based on embedded and top‐mounted (stacked) Ag NW electrodes, respectively, where the embedded electrode contributes to an eightfold greater photocurrent with equivalent dark current performance, as shown in Figure 9c.

#### 5.1.3 Photovoltaic Devices

High‐efficiency solar cells also benefit from MMNN soft TEs.^[^ ^28^, ^29^, ^241^ ^]^ Figure 9d illustrates the usage of Ag micro‐meshes as a soft TE for perovskite solar cells. This device demonstrated higher performance in terms of power conversion efficiency (PCE) and mechanical flexibility when compared to the counterpart employing ITO/PET as the TE.^[^ ^161^ ^]^ The MMNN TEs are expected to have a high aspect ratio in order to enhance electrical conductivity without degrading optical transparency. A very high front‐to‐rear PCE ratio of dye‐sensitized solar cells is made possible by a thick yet narrow Ni micro‐mesh TE.^[^ ^181^ ^]^ It is important to note that large openings in MMNNs should be avoided in photovoltaics since the long diffusion route will make it difficult to collect the charge carriers produced in the active area. A bonus of plasmonic enhancement occurs in solar cells with nano‐mesh‐based soft TEs through processes such as far‐field scattering, near‐field enhancement, and plasmonic energy transfer including hot electron transfer and resonant energy transfer, to improve optical absorption and increase device efficiency.^[^ ^242^, ^243^ ^]^ Although plasmonic enhancement is increased by a small periodicity, the trade‐off between an effective cavity mode and optical transmittance must be taken into account to achieve maximum PCE.^[^ ^244^ ^]^ Figure 9e displays a perovskite solar cell with an Ag nano‐mesh TE that has a distinctive antireflection characteristic and a PCE of up to 17.06%.^[^ ^233^ ^]^ In addition to serving as bottom TEs, MMNNs can also serve as top TEs. For instance, by spray‐coating, Ag NWs can be directly deposited on top of the hole transport layer in an organic solar cell.^[^ ^245^ ^]^ MMNNs are also employed as the top TE in inorganic solar cells to create the metal‐insulator‐semiconductor junction, which has a significantly simplified fabrication and excellent semiconductor passivation, as shown in Figure 9f. A top electrode for uniform charge carrier extraction with great optical transparency is made possible with the closely spaced MMNN TE. Meanwhile, the inverted nano‐pyramid can be patterned using the MMNN electrode as an etch mask to significantly lower the solar cell's reflectance.^[^ ^234^ ^]^

### 5.2 Bioelectronics

Bioelectronic devices are typically implanted and applied to the skin. When it comes to epidermal devices, the superior softness of the electrodes is essential for the device to be placed on the skin in a conformal manner, while high transparency is always desired for optical communication or display purposes. The criteria for the softness of the electrodes in implanted devices is much more stringent since they are attached to surfaces with more intricate topographies.

#### 5.2.1 Epidermal Devices

Skin‐mountable bioelectronics provides the perfect platform for long‐term and real‐time data collection^[^ ^246^ ^]^ or therapy^[^ ^175^, ^191^ ^]^ in the rapidly expanding field of human healthcare monitoring. epidermal electronics with highly conductive, durable, transparent, and breathable electrodes are desired for functional effectiveness and user comforts.^[^ ^186^ ^]^ Superior breathability is crucial for long‐term use of epidermal devices because sweat buildup impairs interfacial conformity and adherence and causes incorrect data measurement.^[^ ^247^ ^]^ Due to their simple fabrication process and high FoM, Ag NWs are the most popular material for epidermal devices,^[^ ^191^, ^207^, ^248^, ^249^ ^]^ while Au^[^ ^250^, ^251^ ^]^ and Cu^[^ ^175^ ^]^ networks are also useful alternatives.

**Figure 10:**
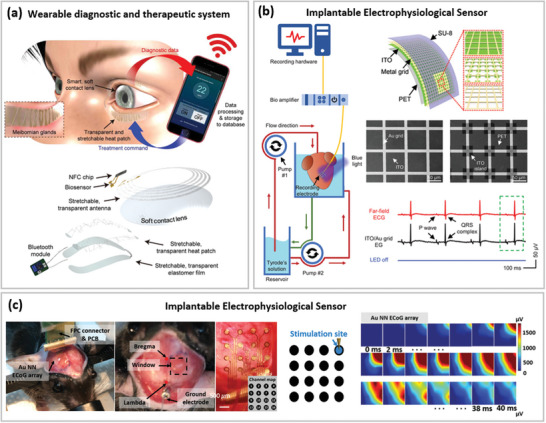
a) Top: schematic illustration of an integrated system consisting of a diagnostic smart lens, an eyelid‐attachable therapeutic heat patch, and wireless communication function via smartphones. Reproduced with permission.^[^ ^192^ ^]^ Copyright 2021, American Association for the Advancement of Science. b) Left: schematic illustration of the Langendorff‐perfusion setup used for electrophysiological recording and optogenetic pacing of ChR2‐expressing mouse hearts; right: schematic illustration (top) and SEM images (middle) of flexible transparent ITO/metal grid hybrid microelectrodes; bottom: far‐field ECG and electrogram recordings of normal sinus rhythm from a reference electrode and an ITO/Au grid hybrid microelectrode with the blue LED turned off, respectively. Reproduced with permission.^[^ ^256^ ^]^ Copyright 2020, Wiley‐VCH GmbH. c) Left: implantation of the Au nano‐mesh electrocorticogram microelectrode array on a cerebral cortex through the cranial window (dashed black line). The location of 16‐channel microelectrodes on the cortical surface is shown with the corresponding channel number; right: spatiotemporal distribution of neural signals was measured and visualized in 2D across the array with light stimulation on the electrode. Reproduced with permission.^[^ ^201^ ^]^ Copyright 2020, Wiley‐VCH GmbH.

Epidermal sensors are often employed to obtain electrocardiogram (ECG) and electromyography (EMG) signals,^[^ ^247^ ^]^ and they can also be utilized as strain sensors ^[^ ^249^ ^]^, tactile sensors,^[^ ^250^ ^]^ temperature detectors,^[^ ^252^ ^]^ and more. ECG sensors based on soft MMNN TE can outperform commercial ones with a superior signal‐to‐noise ratio.^[^ ^249^ ^]^ Furthermore, wireless communication may be achieved by integrating coil‐shaped MMNN‐based TE into the monitoring system.^[^ ^248^ ^]^ Smart contact lenses are a special kind of wearable sensors, which can detect both physical biomarkers from the eyes and chemical biomarkers from tears.^[^ ^253^ ^]^ When they are incorporated with epidermal therapeutic devices via wireless communication, instantaneous diagnosis and corresponding automated treatment can be realized. **Figure** **10**a demonstrates a system integrating a smart contact lens and a skin‐mountable therapeutic device for wireless monitoring and therapy of chronic ocular surface inflammation.^[^ ^192^ ^]^

#### 5.2.2 Implantable Devices

Numerous soft devices have been used in bioelectronics to advance the monitoring of health, the surveillance of disease, and the assessment of therapeutics.^[^ ^183^ ^]^ Research in the fields of neurological and cardiac technology utilizes soft transparent microelectrodes and interconnects that allow for simultaneous optical and electrical examination of biological systems. High‐performance microelectrodes must have high optical transparency for stimulation and recording, exceptional flexibility for conformal contact with highly wrinkled bio‐surfaces, low electrochemical impedance for high signal‐to‐noise ratio, and good biocompatibility. Au and Ag are frequently chosen as microelectrode materials in bioimplantable devices due to their excellent biocompatibility.^[^ ^254^, ^255^ ^]^ The metallic micro‐mesh can function as the electrode with the required optical transmission and electrical impedance, but the effective abiotic/biotic interfacial surface recording area is greatly reduced by the wide opening between metal lines. The integration of additional transparent conductive thin films demonstrates high‐performance electrophysiology and optogenetics for in vivo histology experiments, as shown in Figure 10b.^[^ ^256^ ^]^ Utilizing nano‐meshes, which enable dense networks of high‐resolution sensing and actuation in the spatiotemporal domain, is another technique.^[^ ^257^, ^258^ ^]^ It has been reported that a pure nano‐mesh microelectrode can have great long‐term stability and an electrochemical impedance low enough for photoelectric‐artifact‐free 2D mapping of electrophysiological brain signals with optical stimulation (Figure 10c). While a PEDOT:PSS coating can further reduce the impedance, it degrades the stability in a biological environment.^[^ ^201^ ^]^ Furthermore, by integrating micro‐LEDs into the nano‐mesh soft TEs, colocalized optical modulation and electrophysiological recording can be accomplished.^[^ ^259^ ^]^

### 5.3 Tactile Sensors

Soft TEs also brought about a revolution in established industries such as the billion‐dollar industry for soft human‐machine interfaces (HMIs).^[^ ^260^ ^]^ Flexible transparent touchscreen panels (TSPs), a common type of HMI, are successfully fabricated using MMNN TEs, including both the resistive type^[^ ^7^, ^147^, ^261^, ^262^ ^]^ and the capacitive type.^[^ ^97^, ^177^, ^208^ ^]^ The detection of human movements for human healthcare monitoring is another common use of tactile sensors. In this instance, the new triboelectric sensors^[^ ^198^, ^206^ ^]^ are used in addition to resistive^[^ ^9^, ^188^, ^263^ ^]^ and capacitive^[^ ^264^ ^]^ ones.

#### 5.3.1 Resistive Sensors

**Figure 11:**
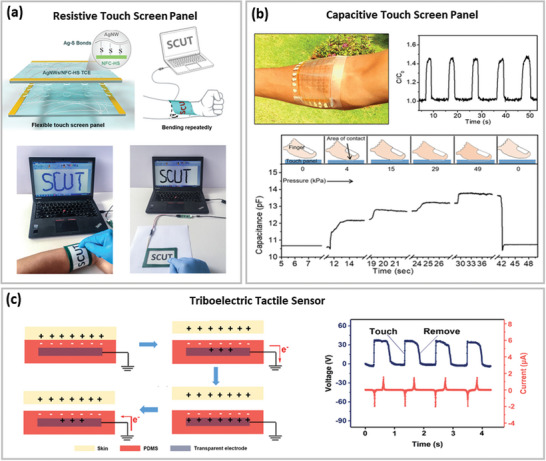
a) Top: schematic illustrations of the structure (left) and measurement system (right) of flexible TSP devices; bottom: device tests on curved (left) and flat (right) conditions. Reproduced with permission.^[^ ^265^ ^]^ Copyright 2021, Elsevier. b) Top: a digital photo of the flexible TPS wrapped over the arm (left) and the capacitance change with cyclic touch stimulus (right); bottom: Capacitance change with finger pressure. Reproduced with permission.^[^ ^177^ ^]^ Copyright 2021, American Chemical Society. c) Left: schematic illustration of the working mechanism for single‐electrode mode TTS; right: real‐time output voltage and output current signals of a hand‐tapping TTS. Reproduced with permission.^[^ ^206^ ^]^ Copyright 2020, Elsevier.

Resistive touch panels, the first generation of tactile sensors, were created in the 1970s. Resistive touch panels, as the name suggests, recognize touch and transfer the signal into a change in resistance. When a high cost‐performance ratio and resolution are priorities, they are the option of choice.^[^ ^265^ ^]^ **Figure** **11**a demonstrates the architecture and performance evaluations of a flexible transparent resistive TSP with Ag NW TEs. The device showed high touch sensitivity, resolution, and mechanical durability in both flat and bending settings, demonstrating the potential of TPSs as wearable HMIs.

#### 5.3.2 Capacitive Sensors

Capacitive TSPs are favored for various integrated systems since they allow for multi‐touch and have less cross‐talk, despite being more costly than their resistive counterparts.^[^ ^266^ ^]^ Figure 11b shows a multifunctional capacitive TSP consisting of Cu micro‐mesh electrodes that are able to detect multiple stimuli including touch, finger proximity, landing, pressure sensing, and temperature monitoring. The superior device performance is attributed to the soft TE's excellent optoelectronic properties, which include its extremely low sheet resistance of 0.79 ohm/sq and high transparency of 90%.^[^ ^177^ ^]^

#### 5.3.3 Triboelectric Sensors

Triboelectric tactile sensors (TTSs) based on single‐electrode nanogenerators take advantage of the coupling effect of triboelectrification and electrostatic induction.^[^ ^8^, ^14^, ^197^ ^]^ Figure 11c illustrates the operation of a single‐electrode mode TTS as well as hand‐tapping open‐circuit voltage and short‐circuit current diagrams.^[^ ^206^ ^]^ TTSs are capable of differentiating between distinct motions like tapping and sliding with the wearable TTS since they are sensitive to not only the magnitude of applied force but also the contacting material with different electronegativity.^[^ ^198^ ^]^

### 5.4 Energy Storage Devices

With the trend of integrating multiple functions into wearable devices that leads to considerable energy consumption, wearable energy storage systems become essential components for next‐generation self‐powered soft transparent electronics.^[^ ^49^ ^]^ Batteries and supercapacitors are two typical representatives of energy storage devices.

#### 5.4.1 Batteries

**Figure 12:**
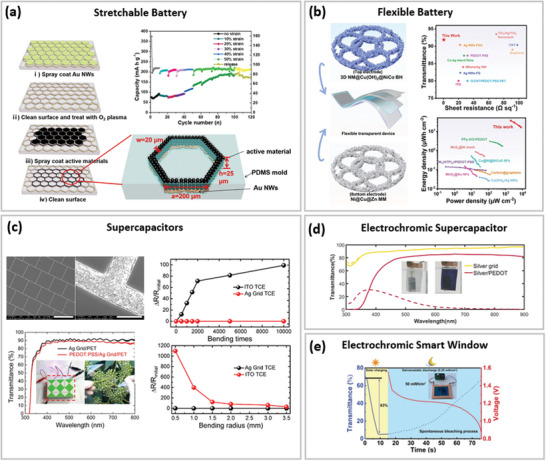
a) Left: schematic illustration of the fabrication process of transparent stretchable Au NW‐based electrodes; right: cyclic performance of the transparent battery. Reproduced with permission.^[^ ^268^ ^]^ Copyright 2021, American Chemical Society. b) Left: schematic illustration of the battery configuration; right: comparison of optoelectronic properties of soft TEs (top) and Ragone plots (bottom) of various batteries. Reproduced with permission.^[^ ^269^ ^]^ Copyright 2022, Wiley‐VCH GmbH. c) Left: SEM images (top) and transparency tests (bottom) of Ag MM‐embedded soft TEs. Right: bending tests of Ag MM/PET and commercial ITO/PET electrodes showing sheet resistance change as functions of bending radius (top) and cycles (bottom). Reproduced with permission.^[^ ^179^ ^]^ Copyright 2017, American Chemical Society. d) Transmittance spectra and digital photos of Ag micro‐mesh soft TEs in colored and bleached states. Reproduced with permission.^[^ ^180^ ^]^ Copyright 2016, Wiley‐VCH GmbH. e) Transmittance spectrum of the zinc anode‐based electrochromic smart window during the solar‐charging and galvanostatic discharge processes. The red line is the corresponding galvanostatic discharge curve, and the inset photo shows an LED lit up by the smart window. Reproduced with permission.^[^ ^270^ ^]^ Copyright 2020, Wiley‐VCH GmbH.

Rechargeable batteries are the most often used power source in our daily lives because of their exceptional features of high energy capacity and endurance.^[^ ^267^ ^]^ Au MM‐based transparent flexible lithium‐ion batteries were created as early as 2011.^[^ ^241^ ^]^ A number of materials and designs have been explored in recent attempts to develop flexible transparent batteries. MMNN soft TEs embedded in PDMS substrates were recently used to create a unique transparent stretchable Zinc ion battery, as illustrated in **Figure** **12**a.^[^ ^268^ ^]^ In comparison to 2D MMNNs, 3‐dimensional (3D) networks are even better candidates for soft TEs in batteries because they allow for a larger mass loading of active materials, which increases energy densities. Using a 3D hierarchical metallic micro‐mesh, Figure 12b shows a high‐performance flexible transparent solid‐state zinc battery with exceptional optoelectronic properties (89.59% transmittance, 0.23 ohm/sq sheet resistance) and mechanical flexibility.^[^ ^269^ ^]^ The completed device showed a tremendous increase in power and energy density above the competition.

#### 5.4.2 Supercapacitors

Lightweight supercapacitors, an energy source known for having a high power density, have been widely researched using paper‐based architectures.^[^ ^183^, ^271^ ^]^ However, the fabrication process is complicated and costs go up due to an additional protective coating of insulating materials that prevents short circuits caused by rough paper surfaces.^[^ ^272^ ^]^ Encapsulating the MMNN TEs in an electroplated MnO_2_ shell is a complex way to address this problem.^[^ ^273^ ^]^ Another issue is transparency. Consequently, extensive research has been conducted to develop transparent supercapacitors using CNTs or graphene as the active layers. However, their specific capacitance is much lower than that of transition‐metal oxides like MnO_2_, and the transparency is compromised when active materials are made thicker to achieve higher areal capacitances.^[^ ^274^ ^]^ MMNNs embedded in soft substrates are the best choice for portable soft transparent supercapacitors because they are thick and narrow and have excellent electrical conductivity and high optical transmittance. Figure 12c shows an ultra‐flexible transparent solid‐state supercapacitor with good mechanical robustness that employs Ag micro‐mesh embedded in a PET substrate.^[^ ^179^ ^]^

Electrochromic components, which modify their optical characteristics in a long‐lasting and reversible way by shifting their oxidation states when subjected to electrical fields, can identify the energy storage status of a transparent supercapacitor, as shown in Figure 12d.^[^ ^180^ ^]^ Electrochromic materials can also be employed in smart windows to store solar energy that has been converted to electrical energy. For instance, a large‐scale flexible transparent zinc anode‐based electrochromic smart window (Figure 12e) was fabricated using a core‐shell structured metal‐mesh electrode. The use of the metal‐mesh anode not only ameliorates the spatially irregular coloration from non‐uniform cation gradient distribution, but also provides an electrochromic performance with exceptional switching times, high optical contrast, and excellent coloring efficiency.^[^ ^270^ ^]^

### 5.5 Other Applications

#### 5.5.1 Heaters

**Figure 13:**
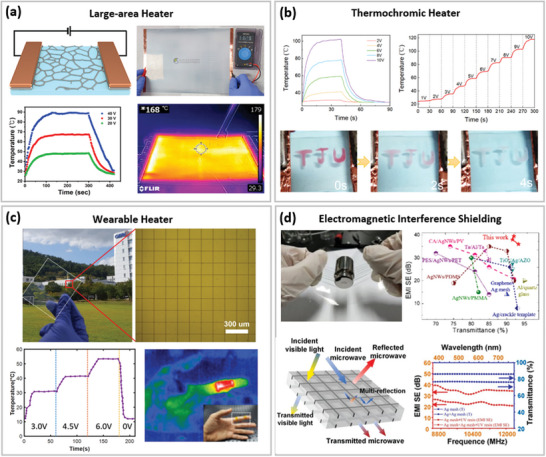
a) Top: schematic (left) and photograph (right) of an A4‐sized heater; bottom: heating‐cooling tests (left) and IR image of the heater at its maximum operating temperature (right). Reproduced with permission.^[^ ^199^ ^]^ Copyright 2021, Elsevier. b) Top: heating‐cooling tests (left) and temperature evolution (right) of the flexible transparent Ag NW heater as a function of applied voltage; bottom: sequential photographs of the heater with letters “TJU” using thermochromic paint. Reproduced with permission.^[^ ^279^ ^]^ Copyright 2021, American Chemical Society. c) Top: photograph (left) and optical micrograph (right) of the Cu mesh/PVA film; bottom: Temperature profiles of the Cu mesh/PVA thermotherapy pad at different input voltages (left) and IR thermal image and photograph of the Cu mesh/PVA heater attached to a finger (right). Reproduced with permission.^[^ ^187^ ^]^ Copyright 2021, Wiley‐VCH GmbH. d) Top: Ni MNs with superior mechanical flexibility and robustness (left) and high transmittance and high EMI SE (right); bottom: Schematic diagram of electromagnetic shielding principle of double‐layer Ag MN (left) and comparison of EMI SE and transmittance between single‐layer and double‐layer Ag meshes. Top: Reproduced with permission.^[^ ^144^ ^]^ Copyright 2023, Elsevier. Bottom: Reproduced with permission.^[^ ^69^ ^]^ Copyright 2019, Optica Publishing Group.

Transparent heaters (THs), a potentially vital element for various devices such as smart windows, displays, actuators, and sensors, can be developed based on Joule heating on the resistance of soft TEs.^[^ ^275^ ^]^ A fast response is desired for heaters, which can be realized by Ag micro‐mesh heaters with proper pattern design.^[^ ^276^ ^]^ Large‐area TEs are required for practical applications such as fog removal on automobile windshields.^[^ ^277^ ^]^ **Figure** **13**a illustrates the development of an A4‐sized large‐area thin‐film heater with Ag NW electrodes, which exhibits remarkable uniformity of heat distribution when powered on thanks to the scalable fabrication of MMNN TEs.^[^ ^199^ ^]^ Another important factor of heaters is the maximal achievable temperature. High‐temperature THs with heating temperature up to 360 °C at 9 V are fabricated with EHD‐printed Ag microgrids.^[^ ^51^ ^]^ Comparable performance can be realized by Ag nano‐mesh heaters, which reach 245 °C at 7 V.^[^ ^278^ ^]^ The temperature can be accurately controlled by varying the applied voltage as illustrated in Figure 13b, and it can be observed using thermochromic materials, whose colors change as the temperature changes.^[^ ^128^, ^279^ ^]^ Similarly, stretchable THs made by embedding the MMNN in elastomers have been reported.^[^ ^25^ ^]^ Investigations are also being conducted on wearable heaters that have a lot of potential for use in on‐skin electronics and medical thermotherapy applications, like the transparent Cu mesh/PVA thin film heater shown in Figure 13c. It responds quickly to thermal input and requires minimal voltage input.^[^ ^187^ ^]^ Moreover, the MMNN heater can incorporate various sensing capabilities to create transparent wearable multipurpose devices.^[^ ^173^, ^194^, ^280^ ^]^

#### 5.5.2 Electromagnetic Interference (EMI) Shielding

Soft electrodes with a strong EMI shielding effect are required because the growing market for electronic devices is causing electromagnetic radiation levels to rise, which is posing a major risk to human health and interfering with electronic equipment. In accordance with the soft TEs' optoelectronic properties, high‐performance flexible transparent EMI films need to be mechanically flexible and possess high optical transmittance as well as high electrical conductivity. For this reason, TE‐based on MMNNs are the best possible candidates for EMI shielding films. Figure 13d demonstrates a single‐layer free‐standing Ni micro‐mesh and a double‐layer embedded Ag micro‐mesh TEs for EMI shielding.^[^ ^69^, ^144^ ^]^ In general, the shielding efficiency can be improved by enhancing electrical conductivity with Ag NW^[^ ^189^ ^]^ or graphene^[^ ^281^ ^]^ coatings, optimizing structural parameters^[^ ^282^ ^]^, or employing multiple layers.^[^ ^283^ ^]^ Randomizing the MMNN pattern for uniform stray light can enhance the optical system's image quality.^[^ ^284^, ^285^ ^]^

## 6 Challenges and Prospects

The performance of MMNN transparent soft electrodes for flexible and stretchable electronic devices presents several key challenges, which can be categorized into four aspects along with corresponding resolutions:

### 6.1 Conductivity‐Transparency Trade‐Off

Achieving high transparency and low sheet resistance simultaneously is a significant challenge. Improved optoelectronic properties can be achieved through the use of high‐aspect‐ratio metallic structures or innovative material selection. Promising candidates for high electrical conductivity include Ag, Cu, and their alloys. However, incorporating hybrid materials such as conductive polymers or carbon‐based materials can offer synergistic advantages in striking the right balance between conductivity and transparency.

### 6.2 Mechanical Durability

Soft electrodes must maintain their electrical and optical properties during mechanical deformation, such as stretching or bending. Optimizing the micro/nanopattern design of metallic networks is essential, involving proper wavy structures to enhance flexibility and stretchability. Interfacial engineering can also be employed to optimize the interface between the metallic network and the substrate, ensuring good adhesion and mechanical integrity to minimize the occurrence of delamination or cracking during operation.

### 6.3 Stability and Reliability

Long‐term stability and reliability are critical for practical use, necessitating resistance against degradation, oxidation, or delamination under various environmental conditions. Progress in materials science is required to develop materials with enhanced stability against oxidation or corrosion.

### 6.4 Scalability and Cost‐Effectiveness

Developing scalable fabrication methods capable of producing large‐area transparent electrodes at a low cost poses a challenge. Many existing techniques are limited to small areas or involve complex and expensive processes. Solution processing and printing techniques generally offer advantages in terms of large‐area coverage and cost‐effective production. However, novel fabrication techniques that are scalable, cost‐effective, and compatible with soft and flexible substrates still need to be developed.

## 7 Concluding Remarks and Outlook

In this study, we evaluated the optoelectronic performance, mechanical properties, and stability of soft TEs based on various material systems. MMNN‐based TEs can simultaneously achieve outstanding electrical conductance and good optical transmittance, outperforming other contenders that struggle with the trade‐off between conductivity and transparency. Besides, MMNN‐based TEs possess outstanding mechanical flexibility and endurance due to the high ductility of metals, which is validated by a variety of applications and implies their unparalleled competitiveness for soft transparent electronics.

This paper reviews both direct and indirect methods for developing MMNN‐based soft TEs. Typically, metallic nanomaterial solutions are used in direct techniques: straightforward direct coating of NW solutions results in MMNNs with serious haze, non‐uniform conductivity, and low conductance; printing technologies using NP solutions have high resolution but poor production efficiency; LDW is a favorable trade‐off between precision and efficiency. Indirect fabrication includes two steps of network patterning and metallization. Deterministic patterning is favored for commercialization in terms of reproducibility, with photolithography and IL being the choices for large‐area micro‐ and nano‐patterning, respectively. Metallization of thin and thick layers can be realized by vacuum deposition and plating methods, respectively. The final phase in creating soft TEs is the transfer of MMNNs, and for this step embedded transfer is favored due to superior protection and flexibility. This article also provides an overview of several MMNN‐based soft TE applications. High‐performance optoelectronic devices taking advantage of the light scattering properties of metallic micro/nanostructures, high‐resolution simultaneous electrical and optical interrogation bioelectronic devices, and advanced self‐powered soft transparent wearables with high‐precision tactile sensors and lightweight energy storage units are all made possible by MMNN‐based TEs.

Finally, we provide a few strategies to expedite the commercialization of MMNN‐based TEs, which focus on material engineering for improvement of optoelectronic properties and stability, better structure design for better mechanical properties, and advanced fabrication technologies for scalability. Moreover, apart from 2D electrodes covered in this review, MMNN‐based TEs show great potential in constructing 3D structural electronics. Solution‐processed in‐situ metallization provides higher throughput and lower cost over conventional 3D printing for the fabrication of stereo electric circuits. In fact, 3D electrodes are currently used for small‐batch production, and abovementioned strategies can also help its mass production and commercialization. MMNN‐based soft TEs hold great promise for continuously advancing wearable technologies and may expand into new applications in the near future.

## Conflict of Interest

The authors declare no conflict of interest.
